# Supplementary material for: Early detection of disease outbreaks and non-outbreaks using incidence data: A framework using feature-based time series classification and machine learning
Source: PLoS Comput Biol. 2025 Feb 13;21(2):e1012782. doi: 10.1371/journal.pcbi.1012782 (PMC11835380; doi:10.1371/journal.pcbi.1012782)
Supplement: S1 Text — (PDF) [file pcbi.1012782.s001.pdf]

Supplementary Materials

In this document, we provide supporting information for the manuscript entitled "Early detection of disease outbreaks and non-outbreaks using incidence data: A framework using feature-based time series classification and machine learning". We start by presenting three stochastic differential equations (SDEs) employed for data simulation. Then we elaborate on two feature extraction libraries (22 statistical features and 5 early warning signal indicators). We further present supplemental results.

1. Data generation

1.1. Model formulation

Within the framework of the SIR model, we incorporated three distinct sources of stochastic variation, namely white noise, multiplicative environmental noise, and demographic noise, to generate the datasets. These models are shown below:

SIR model with White Noise

$$\begin{aligned} dS &= \Lambda dt - \beta(t)SIdt - \mu Sdt + \sigma_1 dW_1 \\ dI &= \beta(t)SIdt - \alpha Idt - \mu Idt + \sigma_2 dW_2 \end{aligned} \tag{1}$$

where σ_1 and σ_2 are noise intensity and $W_i(t)$, $i = 1, 2$, are the Wiener process.

SIR model with Multiplicative Environmental Noise

$$\begin{aligned} dS &= \Lambda dt - \beta SIdt - \mu Sdt + \sigma_1 SdW_1 \\ dI &= \beta SIdt - \alpha Idt - \mu Idt + \sigma_2 IdW_2 \end{aligned} \tag{2}$$

where σ_1 and σ_2 are noise intensity and $W_i(t)$, $i = 1, 2$, are the Wiener process.

SIR model with Demographic Noise

$$\begin{aligned} dS(t) &= \Lambda dt - \beta(t)S(t)I(t)dt - \mu S(t)dt + \frac{a(t) + d(t)}{e(t)}dW_1(t) + \frac{b(t)}{e(t)}dW_2(t), \\ dI(t) &= \beta(t)S(t)I(t)dt - \alpha I(t)dt - \mu I(t)dt + \frac{b(t)}{e(t)}dW_1(t) + \frac{c(t) + d(t)}{e(t)}dW_2(t), \end{aligned} \tag{3}$$

where $a(t) = \Lambda + \beta(t)S(t)I(t) + \mu S(t)$, $b(t) = -\beta(t)S(t)I(t)$, $c(t) = \beta(t)S(t)I(t) + \alpha I(t) + \mu I(t)$, $d(t) = \sqrt{a(t)c(t) - b^2(t)}$, $e(t) = \sqrt{a(t) + c(t) + 2d(t)}$, and $W_i(t)$, $i = 1, 2$, are the Wiener process.

We simulate 14400 time series from each of these three SDEs, with half exhibiting transcritical bifurcation (outbreak) and the rest showing null bifurcation (normality). Additionally, we compile one dataset, MixedN, by randomly choosing 2400 time series for each scenario from the other three datasets. The summary is provided in Table A:

		WhiteN	EnvN	DemN	MixedN
**Training**	Transcritical	6000	6000	6000	6000
	Null	6000	6000	6000	6000
**Testing**	Transcritical	1200	1200	1200	1200
	Null	1200	1200	1200	1200

Table A. Summary of Training set and withheld Testing set.

1.2. Simulations with different amplitudes of noise

We plotted 20 randomly selected replicates of the simulation data for each scenario, with each replicate shown in a different shade in the corresponding panel. One can see that the simulation exhibits different shapes by adding different types of noise to the SIR model.

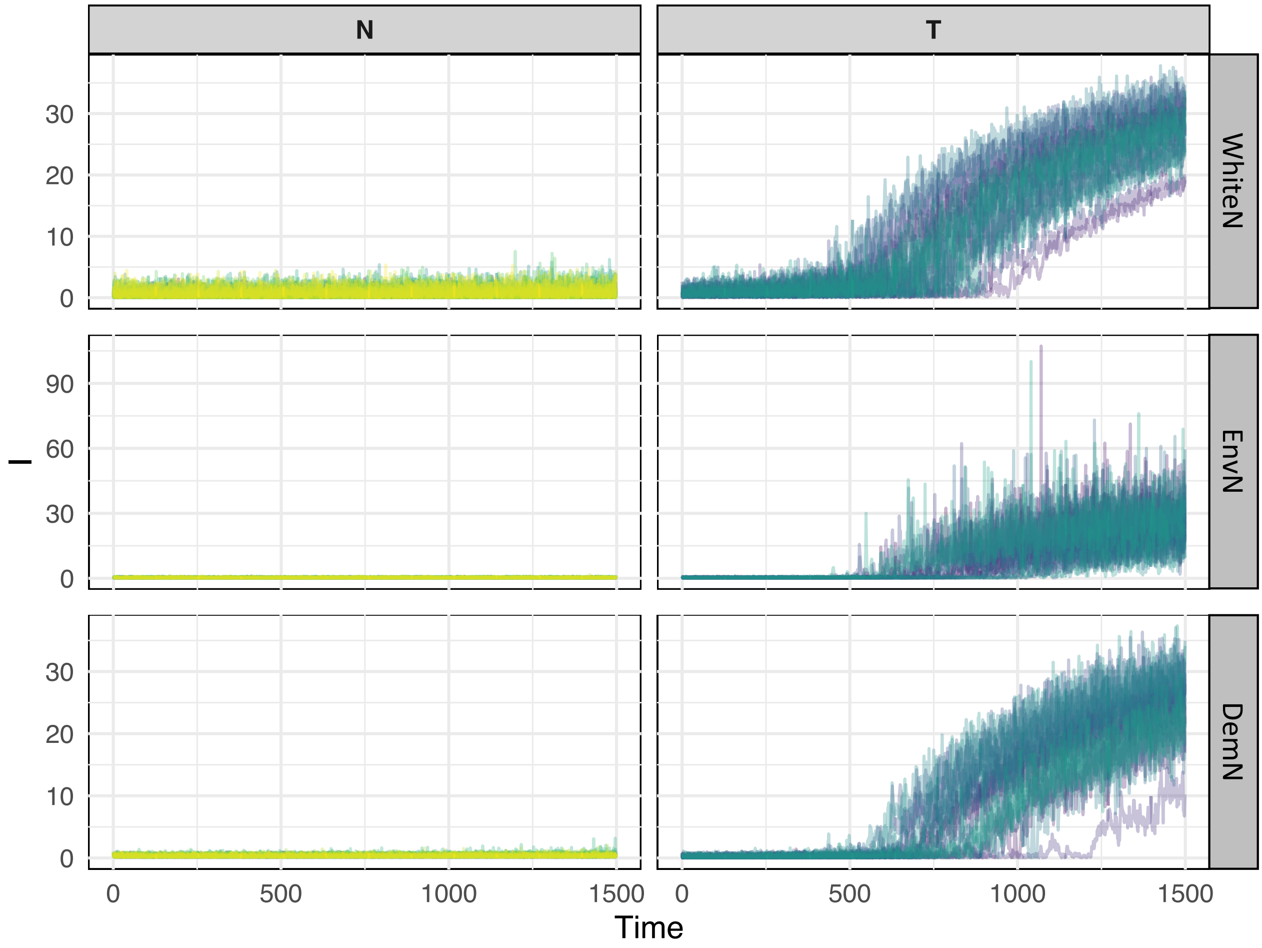

Fig A. Simulation of infected individuals I of transcritical (”T”) and null (”N”) of the SIR model with white noise (WhiteN), multiplicative environmental noise (EnvN), and demographic noise (DemN).

2. 22 statistical features and Mann-Whitney U Test results over synthetic data

In this section, we present detailed descriptions of 22 statistical features [1] in Table B. Then the box plots of each feature computed from "T" and "N" samples are provided in Figs B-E. Since the feature distributions are skewed in most cases, we perform the Mann-Whitney U Test, a non-parametric that does not assume specific data distribution, to compare the differences between each feature of "T" and "N" samples.

No.	Feature	Description
1	DN_HistogramMode_5	Mode of z-scored distribution (5-bin histogram)
2	DN_HistogramMode_10	Mode of z-scored distribution (10-bin histogram)
3	CO_f1ecac	First $1/e$ crossing of autocorrelation function
4	CO_FirstMin_ac	First minimum of autocorrelation function
5	CO_HistogramAMI_even_2_5	Automutual information, $m = 2$, $\tau = 5$
6	CO_trev_1_num	Time-reversibility statistic, $\left\langle (x_{t+1} - x_t)^3 \right\rangle_t$
7	MD_hrv_classic_pnn40	Proportion of successive differences exceeding 0.04σ [2]
8	SB_BinaryStats_mean_longstretch1	Longest period of consecutive values above the mean
9	SB_TransitionMatrix_3ac_sumdiagcov	Trace of covariance of transition matrix between symbols in 3-letter alphabet
10	PD_PeriodicityWang_th0_01	Periodicity measure of [3]
11	CO_Embed2_Dist_tau_d_expfit_meandiff	Exponential fit to successive distances in 2-d embedding space
12	IN_AutoMutualInfoStats_40_gaussian_fmmi	First minimum of the automutual information function
13	FC_LocalSimple_mean1_tauresrat	Change in correlation length after iterative differencing
14	DN_OutlierInclude_p_001_mdrmd	Time intervals between successive extreme events above the mean
15	DN_OutlierInclude_n_001_mdrmd	Time intervals between successive extreme events below the mean
16	SP_Summaries_welch_rect_area_5_1	Total power in lowest fifth of frequencies in the Fourier power spectrum
17	SB_BinaryStats_diff_longstretch0	Longest period of successive incremental decreases
18	SB_MotifThree_quantile_hh	Shannon entropy of two successive letters in equiprobable 3-letter symbolization
19	SC_FluctAnal_2_rsrangefit_50_1_logi_prop_r1	Proportion of slower timescale fluctuations that scale with DFA (50% sampling)
20	SC_FluctAnal_2_dfa_50_1_2_logi_prop_r1	Proportion of slower timescale fluctuations that scale with linearly rescaled range fits
21	SP_Summaries_welch_rect_centroid	Centroid of the Fourier power spectrum
22	FC_LocalSimple_mean3_stderr	Mean error from a rolling 3-sample mean forecasting

Table B. The catch22 feature set spans a diverse range of time series characteristics representative of the diversity of interdisciplinary methods for time-series analysis [1].

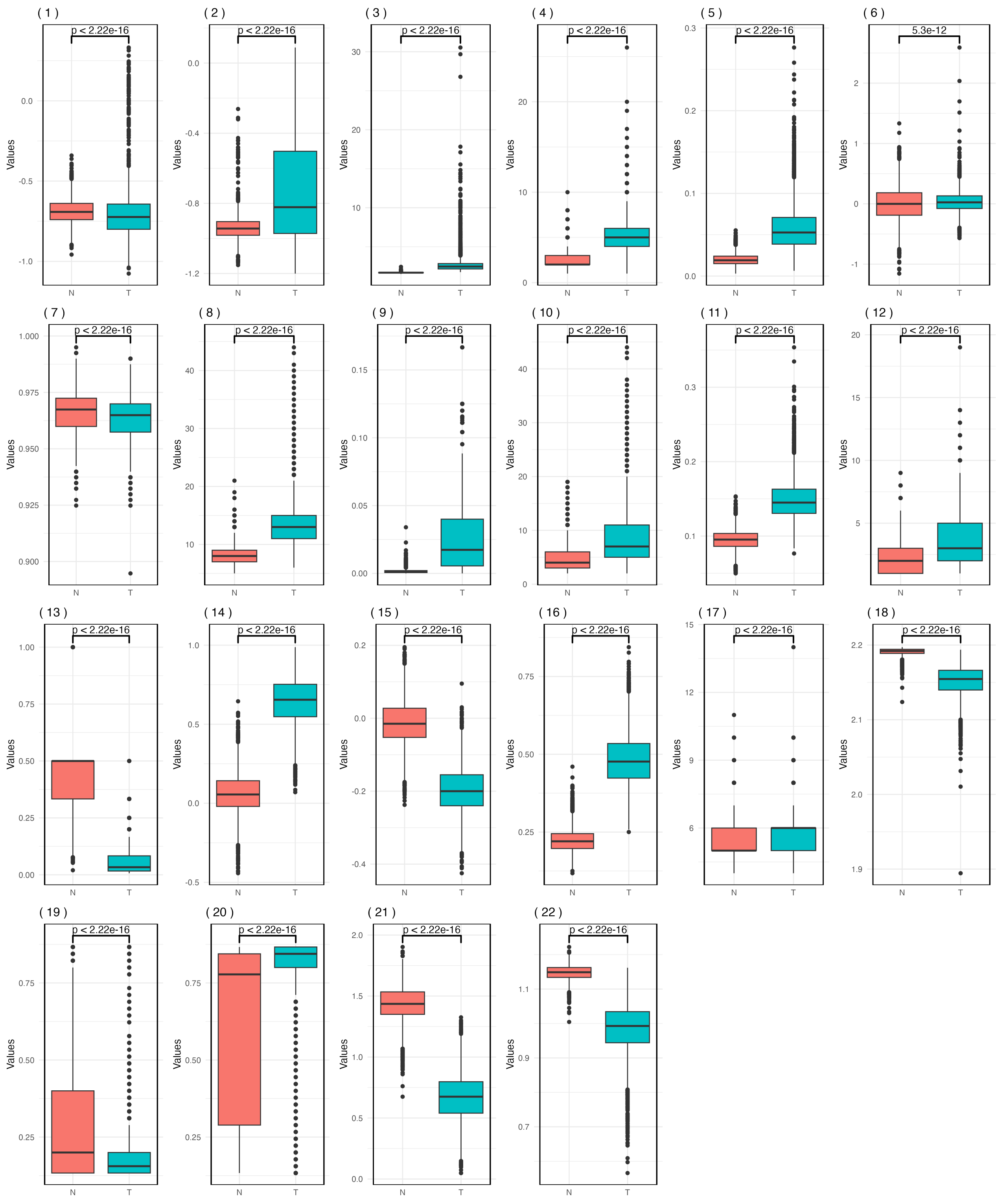

Fig B. Features computed for the two types of generated data (white noise). N indicates null bifurcation data and T indicates transcritical bifurcation data. There are 22 time series features, see Table B for details. P-Values for 22 Features using Mann-Whitney U Test.

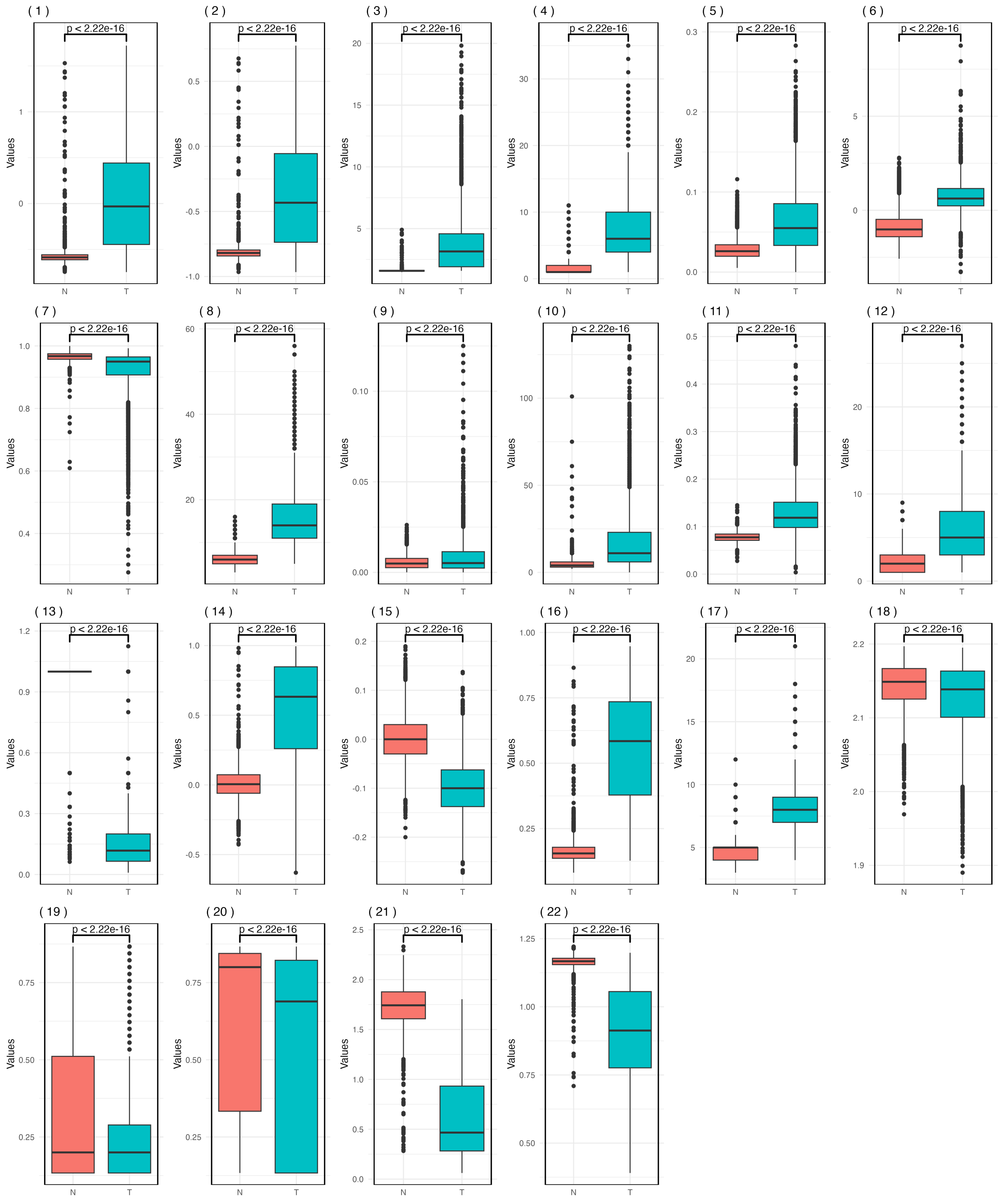

Fig C. Features computed for the two types of generated data (environmental noise). N indicates null bifurcation data and T indicates transcritical bifurcation data. There are 22 time series features, see Table B for details. P-Values for 22 Features using Mann-Whitney U Test.

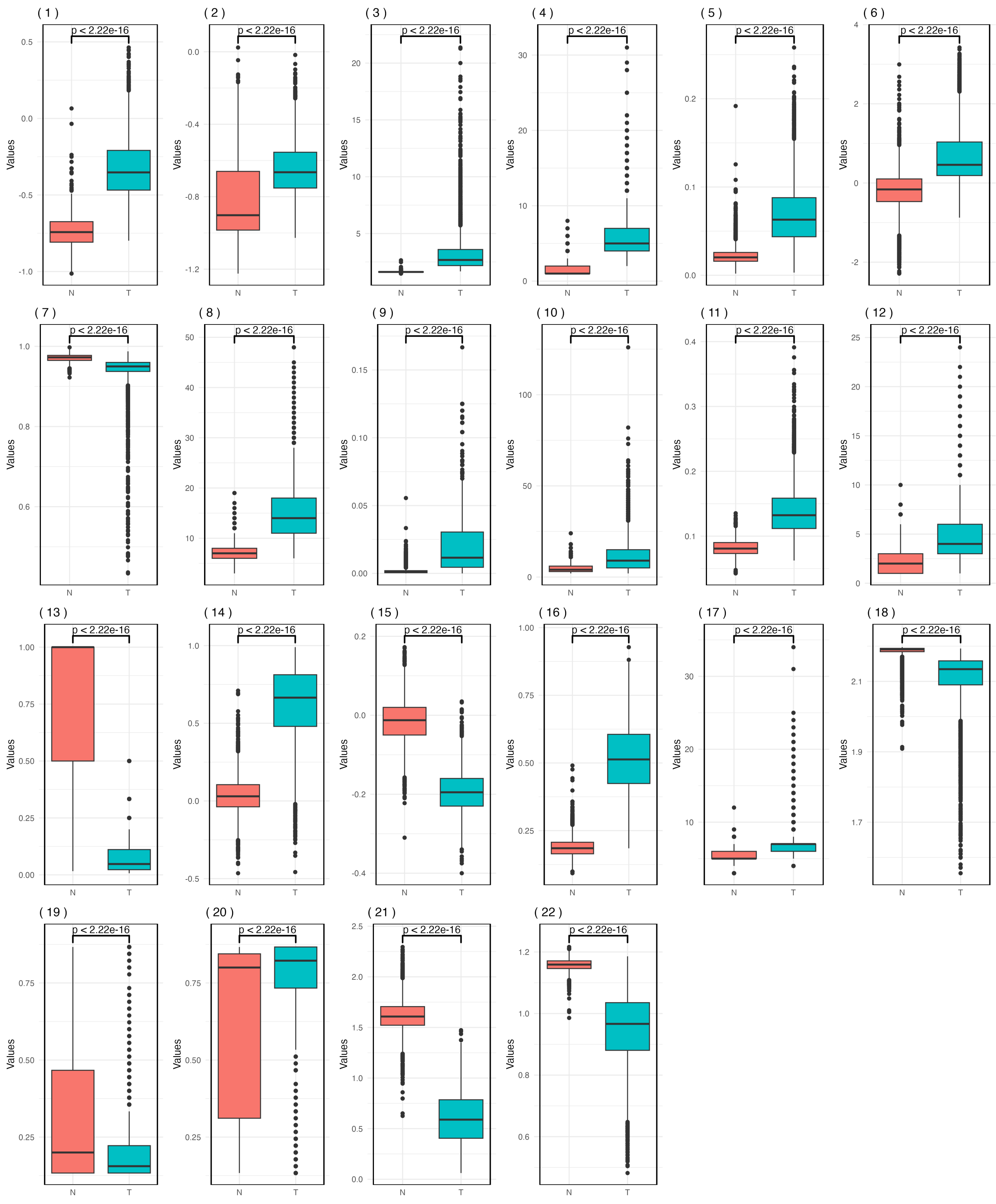

Fig D. Features computed for the two types of generated data (demographic noise). N indicates null bifurcation data and T indicates transcritical bifurcation data. There are 22 time series features, see Table B for details. P-Values for 22 Features using Mann-Whitney U Test.

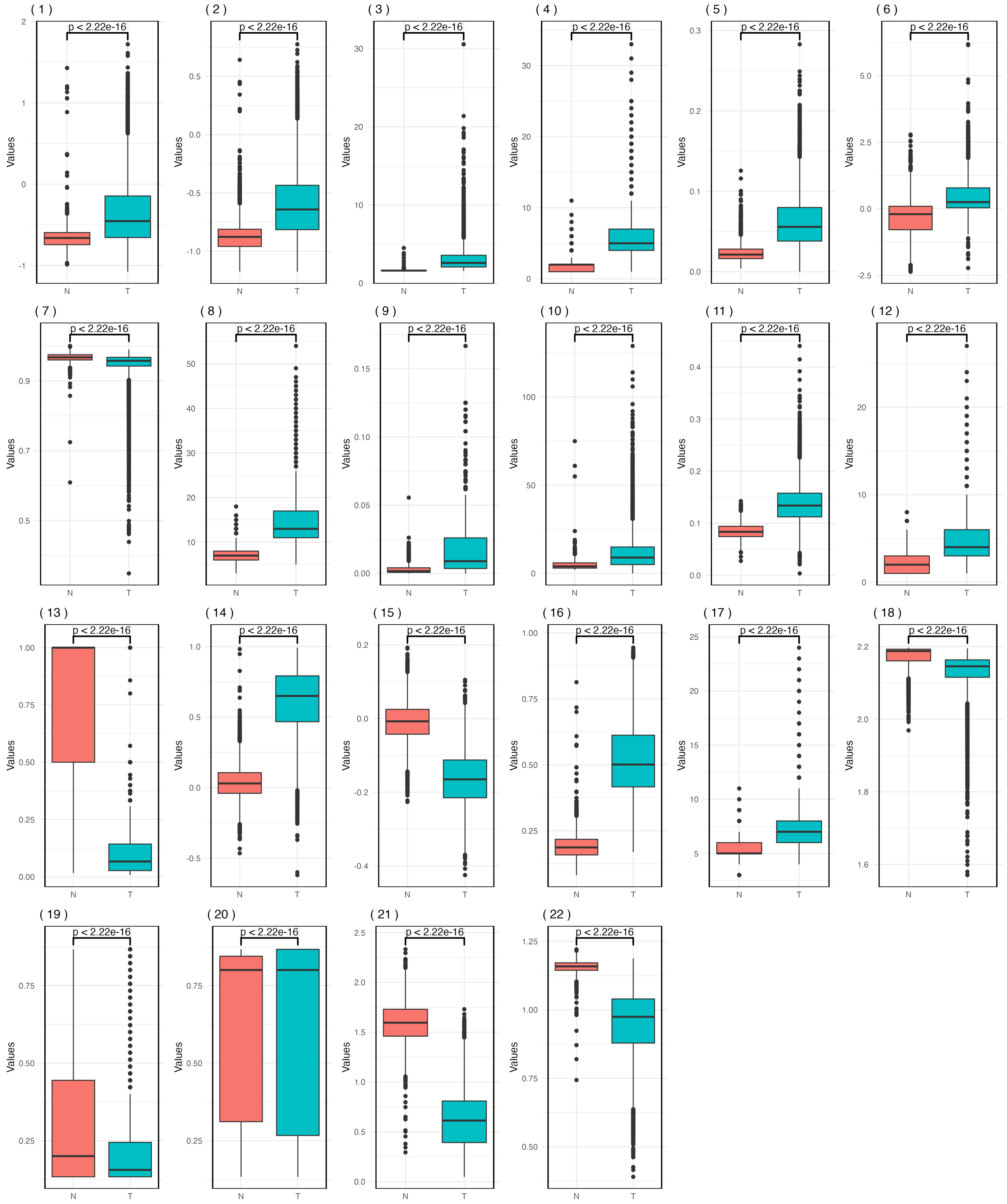

Fig E. Features computed for the two types of generated data (mixed noise). N indicates null bifurcation data and T indicates transcritical bifurcation data. There are 22 time series features, see Table B for details. P-Values for 22 Features using Mann-Whitney U Test.

3. 5 early warning signal indicators and Mann-Whitney U Test results over synthetic data

Here, we present detailed descriptions of 5 early warning signal indicators in Table C. Then the box plots of each feature computed from “T” and “N” samples are provided in Figs F-I. Similar to 22SF, we perform the Mann-Whitney U Test on each feature of ”T” and ”N” samples, as the features do not follow a normal distribution.

Indicator	Formula	Trend	Reference
Standard Deviation (SD)	$\sigma(X) = \sqrt{\frac{\sum_{i=1}^{N}(x_i-\bar{x})^2}{N-1}}$	Increase	[4]
Coefficient of Variation (CV)	$\mathrm{CV} = \frac{\sigma}{\bar{x}} \times 100\%$	Increase	[4]
Autocorrelation at Lag1 (AR1)	$AR^{(1)}(X) = \frac{1}{N-1}\sum_{i=1}^{N}\left(\frac{x_i-\bar{x}}{\sigma_x}\right)\left(\frac{y_i-\bar{y}}{\sigma_y}\right)$	Increase	[5]
Skewness	$S(X) = \frac{\sum_{i=1}^{N}(x_i-\bar{x})^3}{N\sigma(X)^3}$	Increase or Decrease	[6]
Kurtosis	$\mathrm{Kurtosis} = \frac{\frac{1}{N}\sum_{i=1}^{N}(x_i-\bar{x})^4}{\left(\frac{1}{N}\sum_{i=1}^{N}(x_i-\bar{x})^2\right)^2}$	Increase	[7]

Table C. Description of 5 early warning indicators (5EWSI). Time series X containing $N(=400)$ elements labeled x_i. Here $\bar{x}$ denotes the mean of x and y_i are the elements of a time series that is generated by shifting X by 1, so to that $y_i = x_{i-1}$.

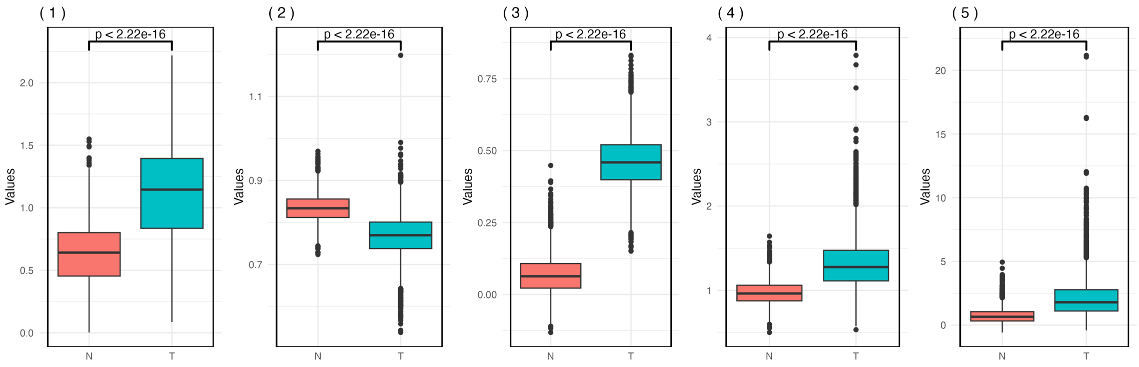

Fig F. EWS indicators computed for the two types of generated data (white noise). N indicates null bifurcation data and T indicates transcritical bifurcation data. There are 5 EWS indicators, see Table C for details. P-Values for 5EWSI using Mann-Whitney U Test.

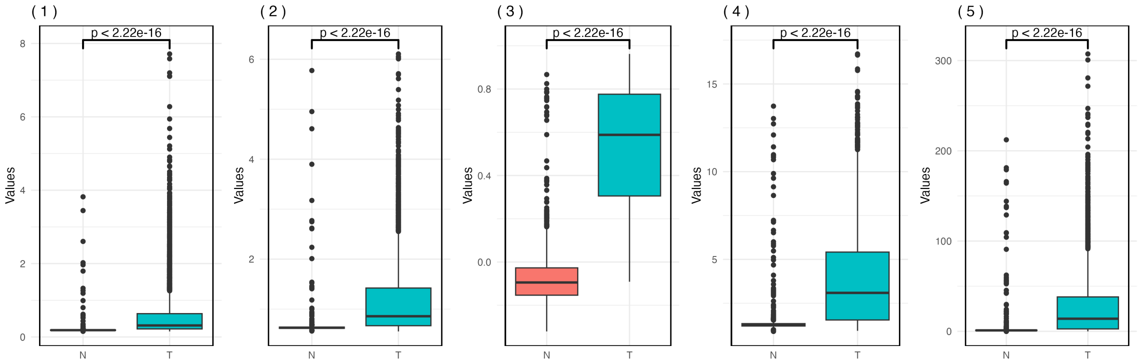

Fig G. EWS indicators computed for the two types of generated data (environmental noise). N indicates null bifurcation data and T indicates transcritical bifurcation data. There are 5 EWS indicators, see Table C for details. P-Values for 5EWSI using Mann-Whitney U Test.

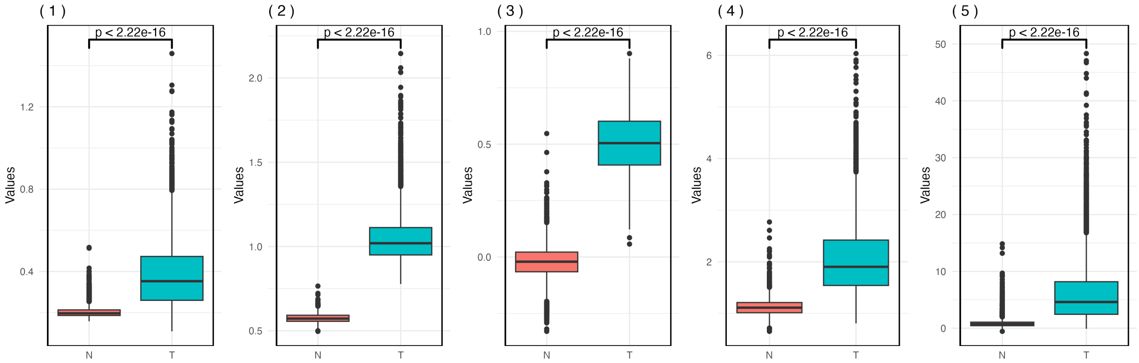

Fig H. EWS indicators computed for the two types of generated data (demographic noise). N indicates null bifurcation data and T indicates transcritical bifurcation data. There are 5 EWS indicators, see Table C for details. P-Values for 5EWSI using Mann-Whitney U Test.

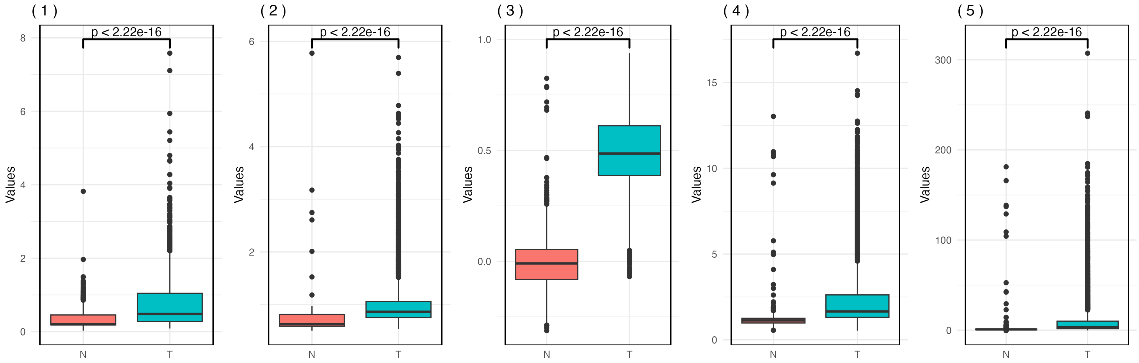

Fig I. EWS indicators computed for the two types of generated data (mixed noise). N indicates null bifurcation data and T indicates transcritical bifurcation data. There are 5 EWS indicators, see Table C for details. P-Values for 5EWSI using Mann-Whitney U Test.

4. t-SNE visualization of statistical features

To further assess the separability of the two classes based on input features, we generated t-distributed stochastic neighbour embedding (t-SNE) plots, shown in Supplementary Figure J. These plots visually confirm the distinct separability of the two classes, supporting the near-perfect classification results on the withheld testing set.

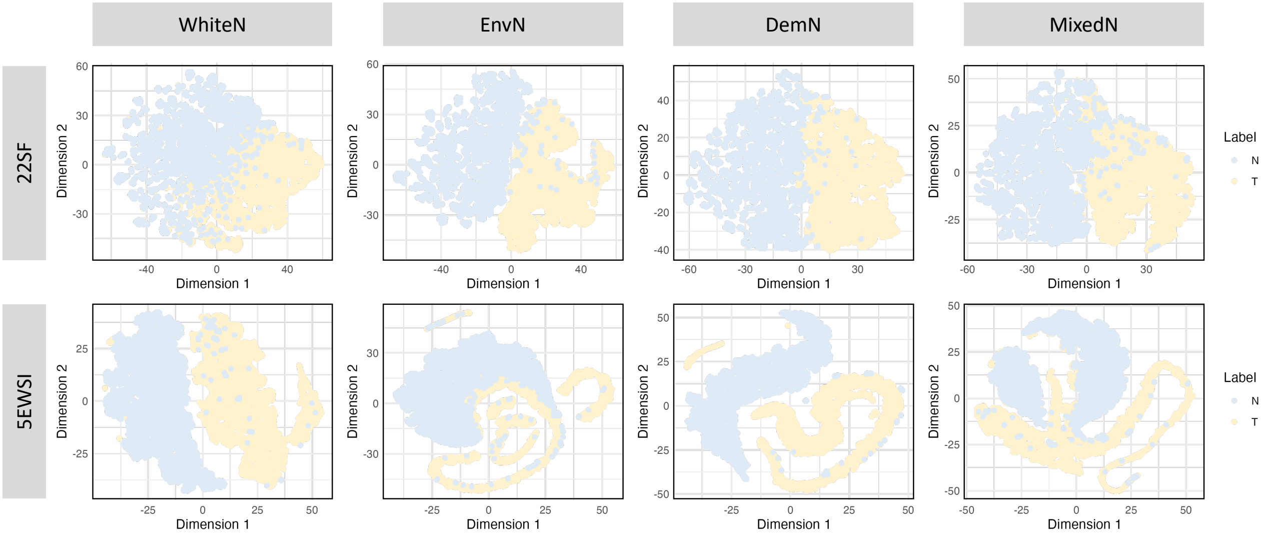

Fig J. t-SNE visualization of statistical features derived from four datasets. Each panel contains a total of 12, 000 samples, with 6, 000 labeled as ”T” and 6, 000 labeled as ”N”.

5. Performance on withheld testing set

Evaluation	Model	22SF			
		WhiteN	EnvN	DemN	MixedN
Accuracy	GBM	0.9963 (±0.0024)	0.9921 (±0.0035)	0.9975 (±0.0020)	0.9958 (±0.0026)
	LRM	0.9963 (±0.0024)	0.9917 (±0.0036)	0.9979 (±0.0018)	0.9933 (±0.0032)
	KNN	0.9954 (±0.0027)	0.9904 (±0.0039)	0.9992 (±0.0011)	0.9971 (±0.0022)
	SVM	0.9971 (±0.0022)	0.9917 (±0.0036)	0.9979 (±0.0018)	0.9938 (±0.0031)
AUC	GBM	0.9999 (±0.0004)	0.9991 (±0.0012)	1 (±0.0000)	0.9998 (±0.0006)
	LRM	1 (±0.0000)	0.9993 (±0.0011)	1 (±0.0000)	0.9996 (±0.0008)
	KNN	0.9995 (±0.0009)	0.9956 (±0.0027)	1 (±0.0000)	0.9995 (±0.0009)
	SVM	1 (±0.0000)	0.9993 (±0.0011)	1 (±0.0000)	0.9995 (±0.0009)
**Evaluation**	**Model**	**5EWSI**			
		WhiteN	EnvN	DemN	MixedN
Accuracy	GBM	0.9988 (±0.0014)	0.9650 (±0.0074)	0.9996 (±0.0008)	0.9796 (±0.0057)
	LRM	0.9992 (±0.0011)	0.9604 (±0.0078)	1 (±0.0000)	0.9775 (±0.0059)
	KNN	0.9983 (±0.0016)	0.9642 (±0.0074)	0.9996 (±0.0008)	0.9825 (±0.0052)
	SVM	1 (±0.0000)	0.9621 (±0.0076)	1 (±0.0000)	0.9783 (±0.0058)
AUC	GBM	1 (±0.0000)	0.9940 (±0.0031)	1 (±0.0000)	0.9983 (±0.0017)
	LRM	1 (±0.0000)	0.9938 (±0.0032)	1 (±0.0000)	0.9973 (±0.0021)
	KNN	0.9992 (±0.0011)	0.9911 (±0.0038)	1 (±0.0000)	0.9974 (±0.0020)
	SVM	0.9996 (±0.0008)	0.9940 (±0.0031)	1 (±0.0000)	0.9974 (±0.0020)

Table D. Classification performance of 32 trained classifiers on withheld testing sets. 22SF, 22 statistical features; 5EWSI, 5 early warning signal indicators; WhiteN (EnvN, DemN, MixedN), simulated data from SIR model with white noise (multiplicative environmental noise, demographic noise, mixed data from the previous three datasets); AUC, rea-under-the-curve; GBM, gradient boosting machine; KNN, k-nearest neighbor; SVM, support vector machines; and LRM, logistic regression model.

5.1. Simple Decision Tree (SDT) and Gradient Boosting Machine (GBM)

While the simple decision tree (SDT) can provide more interpretable classification results, a more complex algorithm, the gradient boosting machine (GBM), can provide a higher classification accuracy as shown in Table E.

Evaluation	Model	22SF			
		WhiteN	EnvN	DemN	MixedN
Accuracy	SDT	0.9867 (±0.0046)	0.9825 (±0.0052)	0.9958 (±0.0026)	0.9879 (±0.0044)
	GBM	0.9999 (±0.0004)	0.9991 (±0.0012)	1 (±0.0000)	0.9998 (±0.0006)
**Evaluation**	**Model**	**5EWSI**			
		WhiteN	EnvN	DemN	MixedN
Accuracy	SDT	0.9867 (±0.0046)	0.9646 (±0.0074)	0.9996 (±0.0008)	0.9738 (±0.0064)
	GBM	0.9988 (±0.0014)	0.9650 (±0.0074)	0.9996 (±0.0008)	0.9796 (±0.0057)

Table E. Classification accuracy of Simple Decision Tree (SDT) and gradient boosting machine (GBM) on withheld testing sets.

5.2. Importance of statistical features

We recorded the importance of statistical features computed from the simple decision trees, as shown in Fig K. For the 5EWSI features, AR1 and CV consistently exhibit high importance, aligning with expectations from EWS theory. Kurtosis, SD, and Skewness exhibit relatively similar values of importance across the four testing sets. There are only four features out of 22SF, namely CO_f1ecac (#3), FC_LocalSimple_mean3_stderr (#13), SP_Summaries_welch_rect_area_5_1 (#16), and SP_Summaries_welch_rect_centroid (#21), have uniformly high importance across four testing sets. The importance of other features varies depending on the type of testing set.

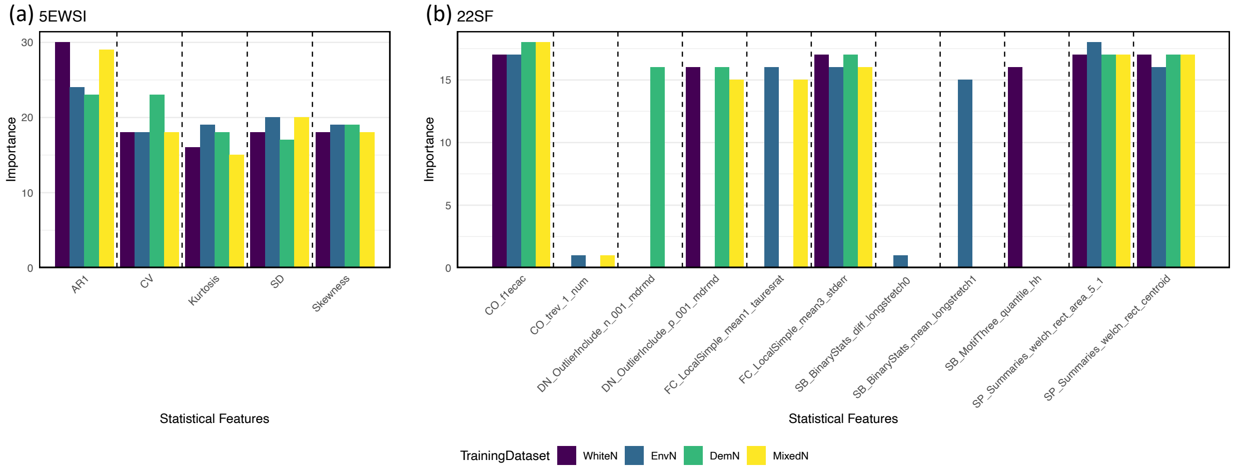

Fig K. The importance of statistical features computed from the simple decision tree.

5.3. Coefficients of logistic regression models (LRM)

Coefficients of logistic regression models (LRM) using 22SF and 5EWSI are presented in Table F and Table G, respectively. Coefficients of variables vary significantly, meaning that these statistical features are

different in simulations from different stochastic models.

No.	Feature	WhiteN	EnvN	DemN	MixedN
0	Intercept	23.97222	−54.99325	121.018738	−5.57765
1	DN_HistogramMode_5	−10.04019	11.78782	8.216708	−0.44300
2	DN_HistogramMode_10	7.03775	−23.13203	−2.405667	1.39135
3	CO_f1ecac	−0.33388	−0.25040	1.166233	−0.87248
4	CO_FirstMin_ac	−0.24283	−0.07706	−0.194280	−0.18338
5	CO_HistogramAMI_even_2_5	−59.31563	−12.41652	−89.935070	−3.23758
6	CO_trev_1_num	0.66380	2.17391	2.964843	3.32243
7	MD_hrv_classic_pnn40	19.14238	−20.16679	−21.127921	8.08251
8	SB_BinaryStats_mean_longstretch1	−0.11836	0.41344	−0.045398	0.23841
9	SB_TransitionMatrix_3ac_sumdiagcov	−19.40297	−4.03937	−51.055396	−3.31577
10	PD_PeriodicityWang_th0_01	−0.02297	−0.02008	0.005524	−0.07171
11	CO_Embed2_Dist_tau_d_expfit_meandiff	12.56613	1.39105	54.186106	32.72535
12	IN_AutoMutualInfoStats_40_gaussian_fmmi	−0.15426	0.08353	−0.142763	0.08974
13	FC_LocalSimple_mean1_tauresrat	−5.73595	−0.86414	−2.024132	−1.02323
14	DN_OutlierInclude_p_001_mdrmd	14.36778	0.41900	5.960529	2.48218
15	DN_OutlierInclude_n_001_mdrmd	−24.32648	−14.59658	−32.769875	−17.03770
16	SP_Summaries_welch_rect_area_5_1	21.71580	−4.22744	−19.625003	8.71233
17	SB_BinaryStats_diff_longstretch0	−0.06463	1.35461	0.426635	1.15949
18	SB_MotifThree_quantile_hh	−22.09094	35.19173	−30.977310	−2.90780
19	SC_FluctAnal_2_rsrangefit_50_1_logi_prop_r1	0.38397	−0.76998	0.662704	−0.93136
20	SC_FluctAnal_2_dfa_50_1_2_logi_prop_r1	−0.57793	0.34980	0.117978	0.61471
21	SP_Summaries_welch_rect_centroid	−14.36507	−6.48014	−12.646018	−5.77143
22	FC_LocalSimple_mean3_stderr	10.30336	−14.18461	−20.597793	−3.43573

Table F. The coefficients of catch22 features of logistic regression models.

No.	Feature	WhiteN	EnvN	DemN	MixedN
0	Intercept	39.3243	6.60514	−134.857	−4.97455
1	Standard Deviation (SD)	4.8576	16.65248	−7.332	−0.60787
2	Coefficient of Variation (CV)	−116.8881	−13.94675	266.171	−4.78762
3	Autocorrelation at Lag1 (AR1)	30.2475	27.07490	−8.878	25.92354
4	Skewness	42.5173	−2.86509	−51.229	3.62273
5	Kurtosis	−4.4873	0.16013	5.099	−0.17710

Table G. The coefficients of 5 early warning indicators of logistic regression models.

6. Classification accuracy results of Rolling window and Expanding window experiments

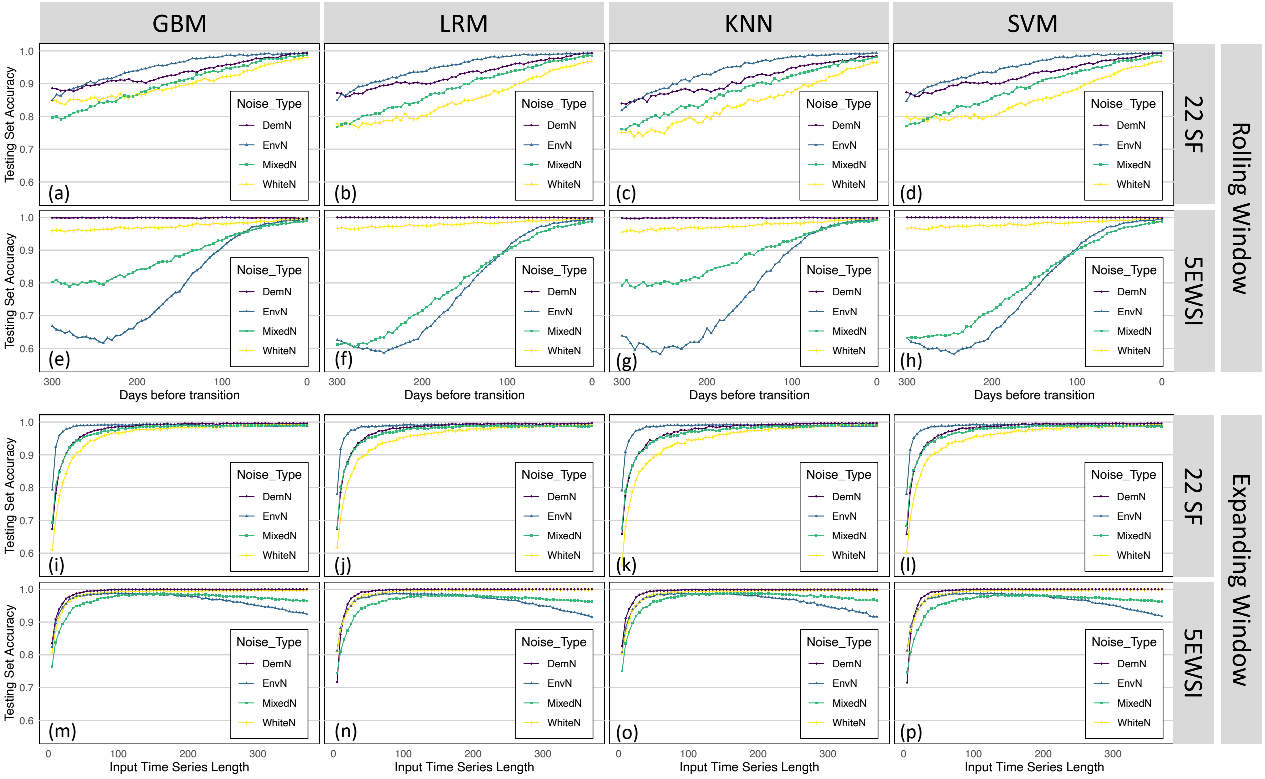

Fig L. Accuracy of synthetic testing sets. We recorded the results of both rolling window and expanding window approaches across 8 datasets and 4 predictive models.

7. Information of COVID-19 incidence data

The SIR model assumes the population is homogeneously mixed, meaning the contact probability between two individuals is identical. However, this assumption may not hold in most country-level studies. Nevertheless, the national-level data is common and relatively accurate owing to its broader research base. We additionally gather the COVID-19 daily incidence data across 18 countries from "Our World in Data" [8] for the case study.

We should acknowledge that the SIR model does not consider the asymptomatic individuals, whereas presymptomatic and asymptomatic cases take more than half of total infection at the outbreak peak, resulting in highly underestimated incidence data [9]. Despite this inherent limitation, our classification approach remains robust and barely affected by the underestimated cases.

ISO Code	Country	Continent	Number of Sliced Sequence	Range of Sequence Length
ARE	United Arab Emirates	Asia	13	16-127
BGD	Bangladesh	Asia	11	14-98
BRA	Brazil	South America	11	14-39
CAN	Canada	North America	7	16-94
CHL	Chile	South America	12	18-97
COL	Colombia	South America	12	14-112
EGY	Egypt	Africa	10	14-73
FIN	Finland	Europe	11	14-69
IND	India	Asia	10	16-149
JPN	Japan	Asia	10	22-96
KOR	South Korea	Asia	10	16-96
MEX	Mexico	North America	11	14-99
MYS	Malaysia	Asia	13	14-74
NGA	Nigeria	Africa	16	14-88
NOR	Norway	Europe	11	14-103
SWE	Sweden	Europe	9	14-87
THA	Thailand	Asia	10	14-136
USA	United States	North America	7	16-67

Table H. ISO Codes and Corresponding Countries with Continents. Sequences containing missing data, or sequence length shorter than 14 are eliminated. As a result, the COVID-19 from 18 countries dataset consists of 194 samples from the original data, with sample lengths ranging from 14 to 149.

8. Mann-Whitney U Test results over COVID-19 data from Edmonton, Canada

We conducted the Mann-Whitney U Test on the empirical data I_{ED} (COVID-19 data from Edmonton, Canada), which is the only empirical dataset that contains both labels. The results are presented in Figure M and Figure N. Some features, such as features (13) and (14) from the 22 statistical features and the kurtosis from the 5 early warning signal indicators, exhibit statistically significant differences between the two labels. For some features with large p-values, we can still see differences with the box plots, likely due to the small sample size. Notably, the $p = 1$ and $p = 0.26$ for variance and AR1, respectively, suggest no significant difference between these two statistics across the two classes. This may explain the poor performance (AUC<0.5) on classification when using these two classic early warning signal indicators, as highlighted in Figure 2 of Chakraborty et al. [10].

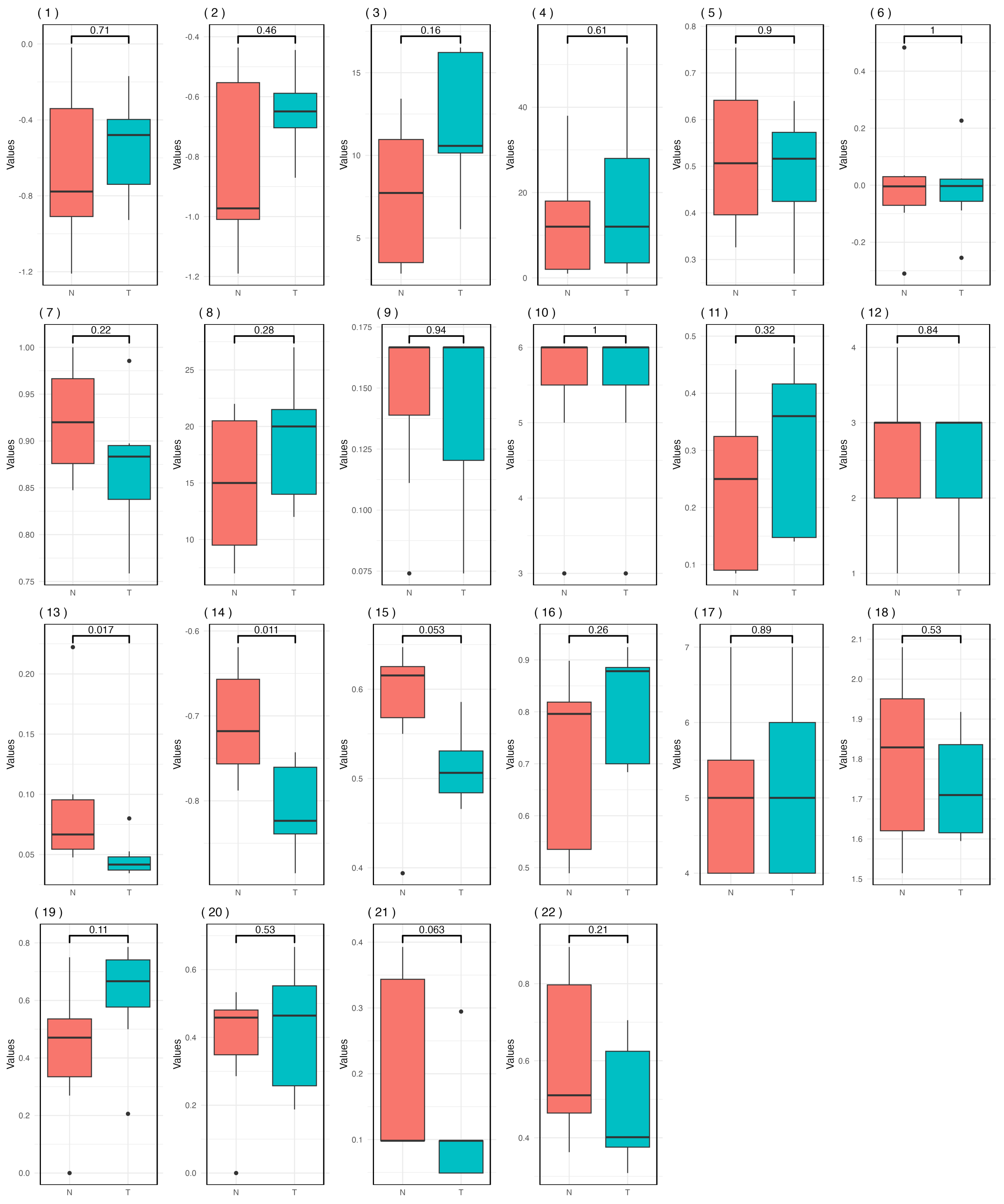

Fig M. Features computed for I_{ED}. N indicates null bifurcation data and T indicates transcritical bifurcation data. There are 22 time series features, see Table B for details. P-Values for 22 Features using Mann-Whitney U Test.

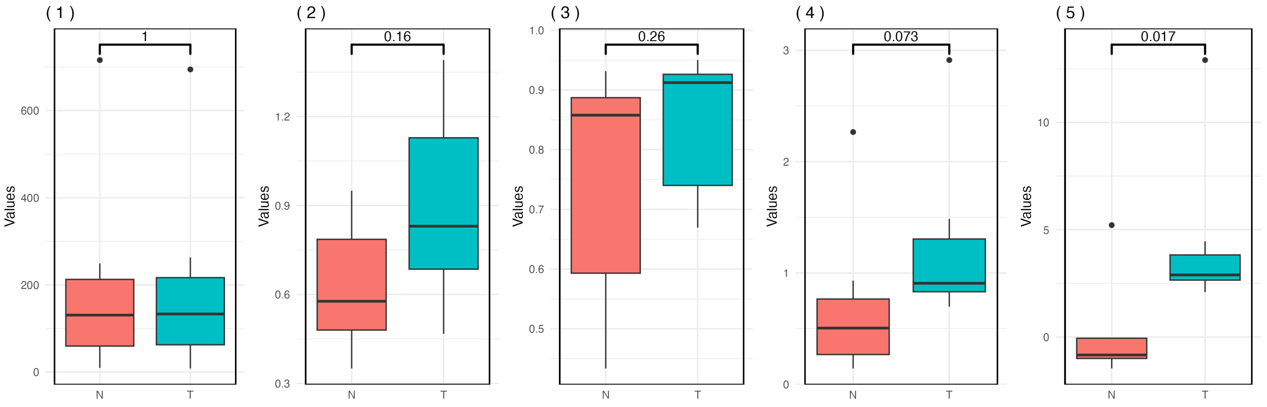

Fig N. EWS indicators computed for I_{ED}. N indicates null bifurcation data and T indicates transcritical bifurcation data. There are 5 EWS indicators, see Table C for details. P-Values for 5EWSI using Mann-Whitney U Test.

9. Effective reproduction number estimation of SARS data

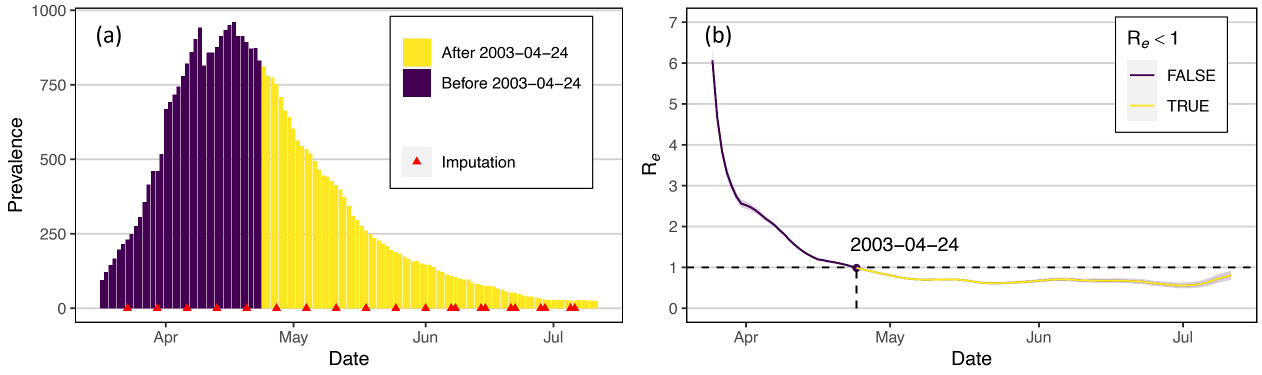

Fig O. Effective reproduction number estimation of SARS data. (a) SARS prevalence data of Hong Kong. The missing data of the original sequence is imputed using `imputeTS`. (b) R_e estimation results through `EpiEstim` with a mean value of 8.4 and standard deviation of 3.8. The shaded region is the 95% confidence interval.

10. Performance on empirical testing sets

Evaluation	Model	22SF			
		WhiteN	EnvN	DemN	MixedN
Accuracy	GBM	0.6392 (±0.0676)	0.3608 (±0.0676)	0.1701 (±0.0529)	0.5258 (±0.0703)
	LRM	0 (±0.0000)	0.0258 (±0.0223)	0.1598 (±0.0516)	0.2010 (±0.0564)
	KNN	0.9588 (±0.0280)	0.7990 (±0.0564)	0.8454 (±0.0509)	0.8454 (±0.0509)
	SVM	0 (±0.0000)	0.0052 (±0.0101)	0.1907 (±0.0553)	0.1495 (±0.0502)
**Evaluation**	**Model**	**5EWSI**			
		WhiteN	EnvN	DemN	MixedN
Accuracy	GBM	0.5619 (±0.0698)	0.8041 (±0.0559)	0.5670 (±0.0697)	0.7371 (±0.0619)
	LRM	1 (±0.0000)	1 (±0.0000)	0 (±0.0000)	0.0103 (±0.0142)
	KNN	1 (±0.0000)	1 (±0.0000)	1 (±0.0000)	1 (±0.0000)
	SVM	1 (±0.0000)	1 (±0.0000)	0 (±0.0000)	0.0103 (±0.0142)

Table I. Classification accuracy of 32 synthetic-data-trained classifiers for COVID-19 Incidence Data of 18 Countries or Regions.

Evaluation	Model	22SF			
		WhiteN	EnvN	DemN	MixedN
Accuracy	GBM	0.4286 (±0.2592)	0.5000 (±0.2619)	0.5000 (±0.2619)	0.5000 (±0.2619)
	LRM	0.5000 (±0.2619)	0.5000 (±0.2619)	0.5000 (±0.2619)	0.5000 (±0.2619)
	KNN	0.5000 (±0.2619)	0.5000 (±0.2619)	0.5000 (±0.2619)	0.5000 (±0.2619)
	SVM	0.5714 (±0.2592)	0.5714 (±0.2592)	0.6429 (±0.2510)	0.5714 (±0.2592)
**Evaluation**	**Model**	**5EWSI**			
		WhiteN	EnvN	DemN	MixedN
Accuracy	GBM	0.5000 (±0.2619)	0.5000 (±0.2619)	0.6429 (±0.2510)	0.5000 (±0.2619)
	LRM	1 (±0.0000)	1 (±0.0000)	0.5714 (±0.2592)	0.5714 (±0.2592)
	KNN	0.5000 (±0.2619)	0.5000 (±0.2619)	0.5000 (±0.2619)	0.5000 (±0.2619)
	SVM	0.5000 (±0.2619)	0.5000 (±0.2619)	0.5000 (±0.2619)	0.5000 (±0.2619)

Table J. Classification accuracy of 32 synthetic-data-trained classifiers for COVID-19 Incidence Data of Edmonton, Canada.

Evaluation	Model	22SF			
		WhiteN	EnvN	DemN	MixedN
Accuracy	GBM	0.8947 (±0.1380)	0.5789 (±0.2220)	0.0526 (±0.1004)	0.8947 (±0.1380)
	LRM	0 (±0.0000)	0 (±0.0000)	0.2105 (±0.1833)	0.1053 (±0.1380)
	KNN	1 (±0.0000)	1 (±0.0000)	1 (±0.0000)	1 (±0.0000)
	SVM	0.0526 (±0.1004)	0 (±0.0000)	0.3158 (±0.2090)	0.1053 (±0.1380)
**Evaluation**	**Model**	**5EWSI**			
		WhiteN	EnvN	DemN	MixedN
Accuracy	GBM	1 (±0.0000)	0.9474 (±0.1004)	0.0526 (±0.1004)	0.9474 (±0.1004)
	LRM	1 (±0.0000)	1 (±0.0000)	0 (±0.0000)	0.2105 (±0.1833)
	KNN	1 (±0.0000)	1 (±0.0000)	0.9474 (±0.1004)	1 (±0.0000)
	SVM	1 (±0.0000)	1 (±0.0000)	0 (±0.0000)	0.2105 (±0.1833)

Table K. Classification accuracy of 32 synthetic-data-trained classifiers for COVID-19 Incidence Data of Singapore.

Evaluation	Model	22SF			
		WhiteN	EnvN	DemN	MixedN
Accuracy	GBM	0.2567 (±0.0247)	0.2283 (±0.0237)	0.9950 (±0.0040)	0.1292 (±0.0190)
	LRM	1 (±0.0000)	1 (±0.0000)	0.6283 (±0.0273)	0.8725 (±0.0189)
	KNN	0 (±0.0000)	0.0008 (±0.0016)	0 (±0.0000)	0 (±0.0000)
	SVM	1 (±0.0000)	1 (±0.0000)	0.2858 (±0.0256)	0.1558 (±0.0205)
**Evaluation**	**Model**	**5EWSI**			
		WhiteN	EnvN	DemN	MixedN
Accuracy	GBM	0 (±0.0000)	0 (±0.0000)	1 (±0.0000)	0 (±0.0000)
	LRM	0.9958 (±0.0037)	1 (±0.0000)	1 (±0.0000)	0.3542 (±0.0271)
	KNN	0 (±0.0000)	0 (±0.0000)	0.0008 (±0.0016)	0 (±0.0000)
	SVM	0 (±0.0000)	0 (±0.0000)	1 (±0.0000)	0.6683 (±0.0266)

Table L. Classification accuracy of 32 synthetic-data-trained classifiers for SARS 2003 Data in Hong Kong.

10.1. Performance on shorter empirical testing sets

Implementation of preventative measures takes time. Hence, we assess the prediction accuracy by allowing one week for preparation. This is achieved by excluding seven data points right before the transition point for all empirical testing samples of COVID-19 data. Results are presented in Table M and Table N.

Evaluation	Model	22SF			
		WhiteN	EnvN	DemN	MixedN
Accuracy	GBM	0.6856 (±0.0653)	0.3041 (±0.0647)	0.1546 (±0.0509)	0.5000 (±0.0704)
	LRM	0.0103 (±0.0142)	0.0155 (±0.0174)	0.1495 (±0.0502)	0.2216 (±0.0584)
	KNN	0.9742 (±0.0223)	0.8247 (±0.0535)	0.8969 (±0.0428)	0.8969 (±0.0428)
	SVM	0.0103 (±0.0142)	0.0052 (±0.0101)	0.1856 (±0.0547)	0.1598 (±0.0516)
**Evaluation**	**Model**	**5EWSI**			
		WhiteN	EnvN	DemN	MixedN
Accuracy	GBM	0.5412 (±0.0701)	0.7629 (±0.0593)	0.5515 (±0.070)	0.6907 (±0.0650)
	LRM	1 (±0.0000)	1 (±0.0000)	0 (±0.0000)	0.0103 (±0.0142)
	KNN	1 (±0.0000)	1 (±0.0000)	1 (±0.0000)	1 (±0.0000)
	SVM	1 (±0.0000)	1 (±0.0000)	0 (±0.0000)	0.0103 (±0.0142)

Table M. Classification accuracy of 32 synthetic-data-trained classifiers for Shorter COVID-19 incidence data of 18 countries or regions.

Evaluation	Model	22SF			
		WhiteN	EnvN	DemN	MixedN
Accuracy	GBM	0.8333 (±0.1826)	0.3889 (±0.2389)	0 (±0.0000)	0.6667 (±0.2310)
	LRM	0 (±0.0000)	0 (±0.0000)	0.3333 (±0.2310)	0.1111 (±0.1540)
	KNN	1 (±0.0000)	1 (±0.0000)	1 (±0.0000)	1 (±0.0000)
	SVM	0 (±0.0000)	0 (±0.0000)	0.2222 (±0.2037)	0.0556 (±0.1123)
**Evaluation**	**Model**	**5EWSI**			
		WhiteN	EnvN	DemN	MixedN
Accuracy	GBM	1 (±0.0000)	1 (±0.0000)	0.0625 (±0.1186)	1 (±0.0000)
	LRM	1 (±0.0000)	1 (±0.0000)	0 (±0.0000)	0.2500 (±0.2122)
	KNN	1 (±0.0000)	1 (±0.0000)	1 (±0.0000)	1 (±0.0000)
	SVM	1 (±0.0000)	1 (±0.0000)	0 (±0.0000)	0.2500 (±0.2122)

Table N. Classification accuracy of 32 synthetic-data-trained classifiers for Shorter COVID-19 Incidence Data of Singapore.

10.2. Performance on larger empirical testing sets

COVID-19 incidence data is often significantly underestimated due to various factors, including presymptomatic and asymptomatic cases that can not be ignored. Although the estimation of the effective reproduction number (R_e) accounts for such underestimation, in practice, people often rely on raw incidence data that can

be potentially smaller than the actual infection numbers. Here, we further test the performance of the classifiers on data expanded fivefold. Accuracy in correctly classifying "larger" testing sets is illustrated in Table O and Table P.

Evaluation	Model	22SF			
		WhiteN	EnvN	DemN	MixedN
**Accuracy**	GBM	0.6837 (±0.0651)	0.3010 (±0.0642)	0.1531 (±0.0504)	0.4949 (±0.070)
	LRM	0.0103 (±0.0141)	0.0155 (±0.0173)	0.1495 (±0.0499)	0.2216 (±0.0581)
	KNN	0.9742 (±0.0222)	0.8247 (±0.0532)	0.8969 (±0.0426)	0.8969 (±0.0426)
	SVM	0.0103 (±0.0141)	0.0052 (±0.0101)	0.1856 (±0.0544)	0.1598 (±0.0513)
**Evaluation**	**Model**	**5EWSI**			
		WhiteN	EnvN	DemN	MixedN
**Accuracy**	GBM	0.5357 (±0.0698)	0.7551 (±0.0602)	0.5459 (±0.0697)	0.6939 (±0.0645)
	LRM	1 (±0.0000)	1 (±0.0000)	0 (±0.0000)	0 (±0.0000)
	KNN	1 (±0.0000)	1 (±0.0000)	1 (±0.0000)	1 (±0.0000)
	SVM	1 (±0.0000)	1 (±0.0000)	0 (±0.0000)	0 (±0.0000)

Table O. Classification accuracy of 32 synthetic-data-trained classifiers for larger COVID-19 incidence data of 18 countries or regions.

Evaluation	Model	22SF			
		WhiteN	EnvN	DemN	MixedN
**Accuracy**	GBM	0.8947 (±0.1380)	0.5789 (±0.2220)	0.0526 (±0.1004)	0.8947 (±0.1380)
	LRM	0 (±0.0000)	0 (±0.0000)	0.2105 (±0.1833)	0.1053 (±0.1380)
	KNN	1 (±0.0000)	1 (±0.0000)	1 (±0.0000)	1 (±0.0000)
	SVM	0.0526 (±0.1004)	0 (±0.0000)	0.3158 (±0.2090)	0.1053 (±0.1380)
**Evaluation**	**Model**	**5EWSI**			
		WhiteN	EnvN	DemN	MixedN
**Accuracy**	GBM	1 (±0.0000)	0.9474 (±0.1004)	0.0526 (±0.1004)	0.9474 (±0.1004)
	LRM	1 (±0.0000)	1 (±0.0000)	0 (±0.0000)	0.1053 (±0.1351)
	KNN	1 (±0.0000)	1 (±0.0000)	1 (±0.0000)	1 (±0.0000)
	SVM	1 (±0.0000)	1 (±0.0000)	0 (±0.0000)	0.1053 (±0.1351)

Table P. Classification accuracy of 32 synthetic-data-trained classifiers for larger COVID-19 Incidence Data of Singapore.

References

[1] C. H. Lubba, S. S. Sethi, P. Knaute, S. R. Schultz, B. D. Fulcher, N. S. Jones, catch22: Canonical time-series characteristics: Selected through highly comparative time-series analysis, Data Mining and Knowledge Discovery 33 (6) (2019) 1821–1852.

[2] J. Mietus, C. Peng, I. Henry, R. Goldsmith, A. Goldberger, The pnnx files: re-examining a widely used heart rate variability measure, Heart 88 (4) (2002) 378–380.

[3] X. Wang, A. Wirth, L. Wang, Structure-based statistical features and multivariate time series clustering, in: Seventh IEEE international conference on data mining (ICDM 2007), IEEE, 2007, pp. 351–360.

[4] S. R. Carpenter, W. A. Brock, Rising variance: a leading indicator of ecological transition, Ecology letters 9 (3) (2006) 311–318.

[5] H. Held, T. Kleinen, Detection of climate system bifurcations by degenerate fingerprinting, Geophysical Research Letters 31 (23) (2004).

[6] V. Guttal, C. Jayaprakash, Changing skewness: an early warning signal of regime shifts in ecosystems, Ecology letters 11 (5) (2008) 450–460.

[7] R. Biggs, S. R. Carpenter, W. A. Brock, Turning back from the brink: detecting an impending regime shift in time to avert it, Proceedings of the National academy of Sciences 106 (3) (2009) 826–831.

[8] E. Mathieu, H. Ritchie, L. Rodés-Guirao, C. Appel, C. Giattino, J. Hasell, B. Macdonald, S. Dattani, D. Beltekian, E. Ortiz-Ospina, M. Roser, Coronavirus pandemic (covid-19), Our World in DataHttps://ourworldindata.org/coronavirus (2020).

[9] S. Raghunath, A. E. Ulloa Cerna, L. Jing, D. P. VanMaanen, J. Stough, D. N. Hartzel, J. B. Leader, H. L. Kirchner, M. C. Stumpe, A. Hafez, et al., Prediction of mortality from 12-lead electrocardiogram voltage data using a deep neural network, Nature medicine 26 (6) (2020) 886–891.

[10] A. K. Chakraborty, S. Gao, R. Miry, P. Ramazi, R. Greiner, M. A. Lewis, H. Wang, An early warning indicator trained on stochastic disease-spreading models with different noises, Journal of the Royal Society Interface 21 (217) (2024) 20240199.
