# Supplementary material for: Early detection of disease outbreaks and non-outbreaks using incidence data: A framework using feature-based time series classification and machine learning
Source: PLoS Comput Biol. 2025 Feb 13;21(2):e1012782. doi: 10.1371/journal.pcbi.1012782 (PMC11835380; doi:10.1371/journal.pcbi.1012782)
Supplement: S2 Text — (PDF) [file pcbi.1012782.s002.pdf]

Supplementary Materials

In this document, we provide supporting information for the manuscript entitled "Early detection of disease outbreaks and non-outbreaks using incidence data: A framework using feature-based time series classification and machine learning".

1. DeLong test results

1.1. Predictive model is fixed

The DeLong test [1] is utilized to compare the difference between the AUC scores of two classifiers. Here, we perform DeLong tests on classifiers derived from eight datasets (i.e. every unique combination of four sets of time series and two feature extraction libraries) with one predetermined predictive model.

When using the same predictive model, AUC scores of classifiers trained from 5EWSI of MixedN (i.e., $M5G$, $M5L$, $M5K$, or $M5S$) are significantly different from those trained from other datasets ($p < 0.05$), with three pairs showing statistically similar AUC scores ($p \leq 0.05$): $M5G$ and $E22G$ ($p = 0.1691$), $M5K$ and $E22K$ ($p = 0.2702$), and $M5K$ and $W5K$ ($p = 0.1125$). Additionally, classifiers trained from 22SF of EnvN (i.e., $E22G$, $E22L$, $E22K$, or $E22S$) exhibit significantly different AUC than classifiers trained from other data across four predictive models ($p < 0.05$ for most cases). There are five exceptions: $M22G$ and $E22G$ ($p = 0.0953$), $M5G$ and $E22G$ ($p = 0.1691$), $M22L$ and $E22L$ ($p = 0.5424$), $M5K$ and $E22K$ ($p = 0.2702$), and $M22S$ and $E22S$ ($p = 0.5320$). The remaining pairs of AUC scores demonstrate statistically identical values, indicating the same performances of their corresponding classifiers.

		**Classifiers**							
		W22G	E22G	D22G	M22G	W5G	E5G	D5G	M5G
**Classifiers**	W22G	1							
	E22G	0.0325	1						
	D22G	0.0951	0.0187	1					
	M22G	0.4003	0.0953	0.1714	1				
	W5G	0.0644	0.0184	0.8616	0.1671	1			
	E5G	<0.0001	<0.0001	<0.0001	<0.0001	<0.0001	1		
	D5G	0.0474	0.0170	0.1464	0.1466	0.2077	<0.0001	1	
	M5G	0.0004	0.1691	0.0002	<0.0001	0.0002	0.0009	0.0002	1

Table A. DeLong Test p-vaLues Results for GBM. $p < 0.001$ (red) indicating very strong evidence against H_0, $p < 0.01$ (orange) strong evidence, $p < 0.05$ (yellow) moderate evidence, $p \geq 0.05$ (green) not significant.

		Classifiers							
		W22L	E22L	D22L	M22L	W5L	E5L	D5L	M5L
Classifiers	W22L	1							
	E22L	0.0311	1						
	D22L	0.2187	0.0240	1					
	M22L	0.1892	0.5424	0.1580	1				
	W5L	0.1133	0.0227	0.3568	0.1514	1			
	E5L	<0.0001	<0.0001	<0.0001	<0.0001	<0.0001	1		
	D5L	0.0858	0.022	0.1653	0.1488	0.3171	<0.0001	1	
	M5L	0.0004	0.0146	0.0004	0.0056	0.0004	0.0059	0.0004	1

Table B. DeLong Test p-vaLues Results for LRM. $p < 0.001$ (red) indicating very strong evidence against H_0, $p < 0.01$ (orange) strong evidence, $p < 0.05$ (yellow) moderate evidence, $p \geq 0.05$ (green) not significant.

		Classifiers							
		W22K	E22K	D22K	M22K	W5K	E5K	D5K	M5K
Classifiers	W22K	1							
	E22K	0.0064	1						
	D22K	0.2617	0.0013	1					
	M22K	0.9666	0.0061	0.2841	1				
	W5K	0.6366	0.0169	0.1582	0.6113	1			
	E5K	<0.0001	0.0116	<0.0001	<0.0001	<0.0001	1		
	D5K	0.2570	0.0012	0.2527	0.2790	0.1558	<0.0001	1	
	M5K	0.0396	0.2702	0.0051	0.0085	0.1125	0.0012	0.0050	1

Table C. DeLong Test p-vaLues Results for KNN. $p < 0.001$ (red) indicating very strong evidence against H_0, $p < 0.01$ (orange) strong evidence, $p < 0.05$ (yellow) moderate evidence, $p \geq 0.05$ (green) not significant.

		Classifiers							
		W22S	E22S	D22S	M22S	W5S	E5S	D5S	M5S
**Classifiers**	W22S	1							
	E22S	0.0216	1						
	D22S	0.1567	0.0157	1					
	M22S	0.2181	0.5320	0.1819	1				
	W5S	0.9879	0.0222	0.4076	0.2196	1			
	E5S	<0.0001	<0.0001	<0.0001	<0.0001	<0.0001	1		
	D5S	0.0860	0.0148	0.2203	0.1760	0.3213	<0.0001	1	
	M5S	0.0004	0.0187	0.0004	0.0069	<0.0001	0.0065	0.0004	1

Table D. DeLong Test p-vaLues Results for SVM. $p < 0.001$ (red) indicating very strong evidence against H_0, $p < 0.01$ (orange) strong evidence, $p < 0.05$ (yellow) moderate evidence, $p \geq 0.05$ (green) not significant.

1.2. *Training data is fixed*

Additionally, we perform DeLong tests on classifiers derived from four predictive models with eight datasets (i.e. every unique combination of four sets of time series and two feature extraction libraries). Eight tables (Table E-L) are provided, each corresponding to a dataset, to depict the p-values.

		**Algorithms**			
		GBM	LRM	KNN	SVM
**Algorithms**	GBM	1			
	LRM	0.3232	1		
	KNN	0.3468	0.2972	1	
	SVM	0.1147	0.9660	0.2963	1

Table E. DeLong Test p-vaLues Results for fixed data: 22SF of White Noise data WhiteN. $p < 0.001$ (red) indicating very strong evidence against H_0, $p < 0.01$ (orange) strong evidence, $p < 0.05$ (yellow) moderate evidence, $p \geq 0.05$ (green) not significant.

		**Algorithms**			
		GBM	LRM	KNN	SVM
**Algorithms**	GBM	1			
	LRM	0.6767	1		
	KNN	0.0048	0.0083	1	
	SVM	0.6266	0.9282	0.0019	1

Table F. DeLong Test p-vaLues Results for fixed data: 22SF of Multiplicative Environmental Noise data EnvN. $p < 0.001$ (red) indicating very strong evidence against H_0, $p < 0.01$ (orange) strong evidence, $p < 0.05$ (yellow) moderate evidence, $p \geq 0.05$ (green) not significant.

		**Algorithms**			
		GBM	LRM	KNN	SVM
**Algorithms**	GBM	1			
	LRM	0.8024	1		
	KNN	0.2698	0.5530	1	
	SVM	0.3819	0.6886	0.7441	1

Table G. DeLong Test p-vaLues Results for fixed data: 22SF of Demographic Noise data DemN. $p < 0.001$ (red) indicating very strong evidence against H_0, $p < 0.01$ (orange) strong evidence, $p < 0.05$ (yellow) moderate evidence, $p \geq 0.05$ (green) not significant.

		Algorithms			
		GBM	LRM	KNN	SVM
Algorithms	GBM	1			
	LRM	0.5244	1		
	KNN	0.3912	0.9565	1	
	SVM	0.2209	0.9683	0.9207	1

Table H. DeLong Test p-vaLues Results for fixed data: 22SF of Mixed Noise data MixedN. $p < 0.001$ (red) indicating very strong evidence against H_0, $p < 0.01$ (orange) strong evidence, $p < 0.05$ (yellow) moderate evidence, $p \geq 0.05$ (green) not significant.

		Algorithms			
		GBM	LRM	KNN	SVM
Algorithms	GBM	1			
	LRM	0.3307	1		
	KNN	0.1575	0.1561	1	
	SVM	0.4571	0.3308	0.1559	1

Table I. DeLong Test p-vaLues Results for fixed data: 5EWSI of White Noise data WhiteN. $p < 0.001$ (red) indicating very strong evidence against H_0, $p < 0.01$ (orange) strong evidence, $p < 0.05$ (yellow) moderate evidence, $p \geq 0.05$ (green) not significant.

		Algorithms			
		GBM	LRM	KNN	SVM
Algorithms	GBM	1			
	LRM	0.8203	1		
	KNN	0.0186	0.0294	1	
	SVM	0.9857	0.2537	0.01674	1

Table J. DeLong Test p-vaLues Results for fixed data: 5EWSI of Multiplicative Environmental Noise data EnvN. $p < 0.001$ (red) indicating very strong evidence against H_0, $p < 0.01$ (orange) strong evidence, $p < 0.05$ (yellow) moderate evidence, $p \geq 0.05$ (green) not significant.

		Algorithms			
		GBM	LRM	KNN	SVM
Algorithms	GBM	1			
	LRM	1	1		
	KNN	1	1	1	
	SVM	1	1	1	1

Table K. DeLong Test p-vaLues Results for fixed data: 5EWSI of Demographic Noise data DemN. Note that DeLong Test of two ROC curves with AUC == 1 has always p.value = 1 and can be misleading. $p < 0.001$ (red) indicating very strong evidence against H_0, $p < 0.01$ (orange) strong evidence, $p < 0.05$ (yellow) moderate evidence, $p \geq 0.05$ (green) not significant.

		Algorithms			
		GBM	LRM	KNN	SVM
Algorithms	GBM	1			
	LRM	0.0174	1		
	KNN	0.2316	0.8783	1	
	SVM	0.0230	0.2333	0.9239	1

Table L. DeLong Test p-vaLues Results for fixed data: 5EWSI of Mixed Noise data MixedN. $p < 0.001$ (red) indicating very strong evidence against H_0, $p < 0.01$ (orange) strong evidence, $p < 0.05$ (yellow) moderate evidence, $p \geq 0.05$ (green) not significant.

References

[1] E. R. DeLong, D. M. DeLong, D. L. Clarke-Pearson, Comparing the areas under two or more correlated receiver operating characteristic curves: a nonparametric approach, Biometrics (1988) 837–845.
