# Supplementary material for: Early detection of disease outbreaks and non-outbreaks using incidence data: A framework using feature-based time series classification and machine learning
Source: PLoS Comput Biol. 2025 Feb 13;21(2):e1012782. doi: 10.1371/journal.pcbi.1012782 (PMC11835380; doi:10.1371/journal.pcbi.1012782)
Supplement: Attachment — Submitted filename: Response to reviewers.pdf [file pcbi.1012782.s003.pdf]

Response to the Editor and Reviewers: PCOMPBIOL-D-24-00668

Dear Editor and Reviewers:

We thank the editor and reviewers for carefully reading and reviewing our manuscript, *Early detection of disease outbreaks and non-outbreaks using incidence data: A framework using feature-based time series classification and machine learning*. We are grateful for the comments and suggestions, and we have incorporated these into the revised version of the manuscript. All the changes have been highlighted in blue in the revised manuscript.

In the following, we include the point-by-point responses to each comment by the reviewers. In this response letter, we use blue to indicate the significantly revised content of the revised manuscript, with corresponding line numbers in orange. We hope this is sufficient for the continuation of the review process and look forward to hearing from you soon.

Yours sincerely,

The Authors

Response to the Editor

Comment

Thanks for the submission! Both reviewers acknowledged that combining machine learning (ML) and dynamical systems approaches to better forecast epidemics is an interesting approach. However, they raised major concerns with the work. Please address the reviewers' comments, especially the ones regarding the uncertainty quantification of model forecasts (in my opinion, a very relevant issue in practical use of the model) and the reliability of the model trained on synthetic datasets (i.e. a common problem for ML models due to lack of large real-world datasets).

Response:

We sincerely appreciate your effort and insightful feedback on our manuscript. We have carefully considered all the suggestions and made the necessary revisions. Please find our detailed point-by-point response below.

In response to concerns regarding the reliability of our models, we expanded the empirical dataset to include two additional COVID-19 datasets from 18 countries and Edmonton, Canada. The same two models consistently achieved high classification accuracies across all the empirical datasets. Importantly, our model outperformed classification models based on early warning signal indicators, as well as deep learning models proposed by Bury et al. [1] and Chakraborty et al.[2], when applied to the Edmonton, Canada COVID-19 dataset—the same dataset tested in their studies. Given that these two models achieved the best performance on the additional datasets and outperformed previously proposed models on the idnetical Edmonton dataset, we are more confident in asserting the practical effectiveness of our models.

Uncertainties come from different sources such as noise, and inadequate knowledge of model structure [3]. Our work has accounted for uncertainties in different ways. (1) We included three types of noise in the SIR model for simulation to mimic the variability and unpredictable factors in real-world data. (2) We assumed the transmission rate parameter β to increase linearly with positive intercepts and slopes following triangular distribution, making the outbreak emergence time random. (3) We evaluated model performance by varying the sequence length and gap using rolling and expanding windows. (4) We tested the scenarios when data did not follow the homogeneity population assumption of the SIR model, and when data was highly underestimated. Results suggest that our models can handle these two scenarios. (5) Additionally, we also provided the 95 confidence interval for all the classification accuracies. Our results already come from several uncertainties included. Nevertheless, uncertainty quantification (UQ) can be one direction of future work, as we added in the ***1. Discussion*** section.

1 Response to reviewer 1

Comment 1.1

The authors propose a machine learning framework to predict disease outbreak. They train machine learning classifiers on simulation data from a stochastic SIR model. They generate a bifurcation class, where the model is taken through a transcritical bifurcation, and a null class, where the model does not pass through a bifurcation. The classifiers are trained on the binary classification problem of distinguishing these classes. They perform with very high accuracy on a withheld simulation test set. When evaluated on real data from Covid and SARS, 2 out of the 32 trained models perform with high accuracy.

This paper is written well, has clean figures and a good, logical structure. The subject of the study—combining machine learning and dynamical systems to predict disease outbreaks—is interesting and timely. The combinatorial analysis of the different classifiers/noise/features is thorough, as well as the investigation with respect to rolling vs. expanding windows. Overall, the analysis of the approach using model simulations is well done.

Response:

We would like to thank the reviewer for thoroughly reviewing the manuscript and providing constructive feedback and suggestions. Below, we address your comments in detail. While this response letter includes the major revisions in blue with orange corresponding line numbers, minor changes making the revised manuscript self-contained are not included here to maintain the readability of the response letter. However, all changes are heightened in blue in the revised manuscript.

Comment 1.2

However, I am not convinced that this approach, in its current state, is effective on real data. Figure 4 shows that the majority of the trained classifiers perform no better than random (an accuracy of around 0.5 when combining the empirical datasets to balance the classes). This would suggest that the simulated training set is not sufficiently representative of what is occurring in the real data. The fact that 2 out of the 32 models perform well is not a sufficient indication that generalizable features have been learned from the training set to predict real disease outbreak—-These models may have scored high by chance, given the number of models tested. I would need to see further evidence of the performance of these two classifiers on real data before agreeing with the final statement of the abstract—that there are "statistical features distinguishing outbreak and non-outbreak. . . in real-world datasets".

Response:

We appreciate the opportunity to clarify and improve our work. The idea of using mathematical simulations to enrich the data for training and predicting disease outbreaks and non-outbreaks is rooted in early warning signal (EWS) theory. When a system approaches a critical transition—such as a disease outbreak in our context—certain statistical features exhibit increasing or decreasing trends. We apply this phenomenon by training models on simulations that are more controllable, allowing the models to learn these trends and improve early-stage outbreak prediction.

However, discrepancies between simulated and empirical data are inevitable. Relying solely on classic early warning signals may fail to indicate imminent transitions in practice [4, 5]. To address this limitation, we integrate early warning signal theory and machine learning algorithms to better predict the occurrence of infectious disease outbreaks. Then we explored several options for each configuration of the proposed framework and therefore formulated a couple of models. We are not

expecting a universally good performance on the empirical datasets. Instead, the performance on the empirical dataset will be the criterion for model selection and provide insights into the optimal configurations.

Therefore we revised the following in the ***1. Introduction***:

In the end, we test the classifiers on real-world COVID-19 incidence data and SARS data to validate their practical applicability. Performance on empirical datasets also serves as the criterion for model selection, as we tested multiple models derived from different framework configurations. (Line 128-130)

We further added the following to the ***2.4. Performance on empirical testing set*** of the manuscript:

Although our models perform well on withheld test sets, we need to further evaluate their practical utility using real-world datasets. Performance on empirical datasets is also the criterion for model selection as it provides insight into the optimal choices for the model of simulation, feature extraction, and the machine learning algorithm within the framework. (Line 216-219)

In our original manuscript, we tested the performance on two empirical datasets: COVID-19 data from Singapore (I_{SG}) and SARS data from Hong Kong (I_{HK}). We found two models exhibiting good performance on classification tasks and we identified these as the best options. However, we acknowledge that concluding the best models based on the performance of only two empirical datasets is insufficiently convincing. To further validate the performance on empirical datasets, we additionally tested the performance of COVID-19 daily data from Edmonton, Canada (I_{ED}) and 18 countries (I_{18}). The performance is shown in Figure 1 (Figure 6 of the revised manuscript). The same two models, $E5L$ and $W5L$, display the best performance across all four datasets. Notably, our model outperformed classification models based on early warning signal indicators, as well as deep learning models proposed by Bury et al. [1] and Chakraborty et al. [2] when applied to the same COVID-19 dataset from Edmonton, Canada, which was also used in their research.

We added the description of two more empirical datasets to ***4.3. Empirical data used for testing*** of the manuscript:

I_{ED} **(# replicates = 14; COVID-19 daily infection data from Edmonton, Canada)** The dataset I_{ED} was obtained from the City of Edmonton's Open Data Portal [6] and includes daily incidence data from March 2020 to July 2023. Similar to I_{SG}, the R_0 was estimated using `R` package `EpiEstim` [7], and subsequences with $R_0 < 0$ and length is larger than 56 (8 weeks) were selected. Note that this empirical dataset and the data processing are identical to the empirical datasets used in [2] for comparison purposes. (Line 440-444)

I_{18} **(# replicates = 194; COVID-19 incidence data from 18 countries)** While the national data are often underestimated (due to presymptomatic and asymptomatic case) and against the homogeneity of the population, we still use the national-level data to test the robustness of the model. To derive the replicates and the corresponding labels, we apply the R_e estimation from Huisman et al. [8] for COVID-19 incidence data, where the assumption is that the incidence data come from symptomatic individuals. Given the typical lag between disease occurrence and data collection, subsequences with lengths shorter than 14 days (i.e., two weeks), or replicates containing missing data are excluded. The final dataset comprises 194 replicates, labeled as "T", encompassing data from 18 countries. Detailed information on the sliced data is provided in Supplementary Table S20. (Line 445-453)

For the SARS data from Hong Kong, we revised the data description as follows: To ensure greater confidence in the R_e values being less than 1 for the subsequences, we designate the sequence after a later date, May 15, 2003, as the null

bifurcation scenario and randomly select 1200 subsets, each with a random length larger than 7, as the "N" replicates in the testing set. (Line 463-464)

We updated the ***Data availability*** of the manuscript:

Synthetic data are available at `https://doi.org/10.5281/zenodo.10967222` [9]. COVID-19 incidence data from Singapore and 18 countries is from Our World in Data [10]. The COVID-19 daily infection data comes from the City of Edmonton's Open Data Portal [6]. The SARS data for Hong Kong were obtained from `https://www.kaggle.com/datasets/imdevskp/sars-outbreak-2003-complete-dataset?resource=download` [11]. (Line 513-517)

Since we have two more empirical testing sets, we revised the ***2.4. Performance on empirical testing set***:

Although our models perform well on withheld test sets, we need to further evaluate their practical utility using real-world datasets. Performance on empirical datasets is also the criterion for model selection as it provides insight into the optimal choices for the model of simulation, feature extraction, and the machine learning algorithm within the framework.

We tested the performance of 32 simulation-trained classifiers (see Figure 2) on four empirical datasets: two at the national level, COVID-19 data from Singapore (I_{SG}) and from 18 countries (I_{18}), and two at the city level, COVID-19 data from Edmonton, Canada (I_{ED}), and SARS data from Hong Kong (I_{HK}). Although violating the assumption of homogeneous population distribution in the SIR model, we utilize I_{18} to evaluate the performance of classifiers on data that deviates from the model assumption. We use the I_{ED} to compare the performance with existing methods. Note that three datasets (I_{SG}, I_{18}, and I_{HK}) solely contain replicates labeled as either "T" or "N", making it impossible to compute AUC scores. As a result, we use the classification accuracy as the performance measurement on testing sets, and the results are presented in Figure 1.

As shown in Figure 6, the performance varies across the four empirical datasets depending on the type of data used for model training, specifically the simulations generated from the SIR model with different types of noise. Notably, there are two models, $E5L$ and $W5L$, that consistently demonstrate high classification accuracy across all empirical datasets, making them the best-performing models among the 32 candidates. This result indicates that computing five classic early warning indicators from the data and using the logistic regression model would provide better classification performance.

For dataset I_{SG}, 17 out of 32 models performed better than random with 16 achieving an accuracy of 0.9 or higher. For dataset I_{18}, 18 models achieved an accuracy above 0.5, but only 9 models exceeded 0.9. Although I_{SG} and I_{18} are both national-level datasets, we observed generally lower accuracy on I_{18} compared to I_{SG}, which is consistent with our rationale that Singapore data is more suitable for SIR model assumptions. The performance of I_{18}, I_{SG} and I_{HK}, or in other words, between replicates solely labeled as "T" (outbreaks) or "N" (non-outbreaks) seem to be complementary. Most classifiers performing well in classifying "T" replicates fail to predict replicates labeled as "N", except for two classifiers, E5L and W5L, achieving an accuracy of 1 (± 0.0000) in out-of-sample empirical data classification tasks. For dataset I_{ED}, which is also used in Chakraborty et al. [2] as the empirical testing set, the best two models (AUC = 1) outperformed the models trained by classic early warning signals (AUC < 0.5), the deep learning model proposed by Bury. et al [1] (AUC < 0.5), and Chakraborty. et al [2] model (AUC = 0.71). Note that the error bar for I_{ED} is significantly larger compared to the other three datasets as its sample size is much smaller. (Line 216-245)

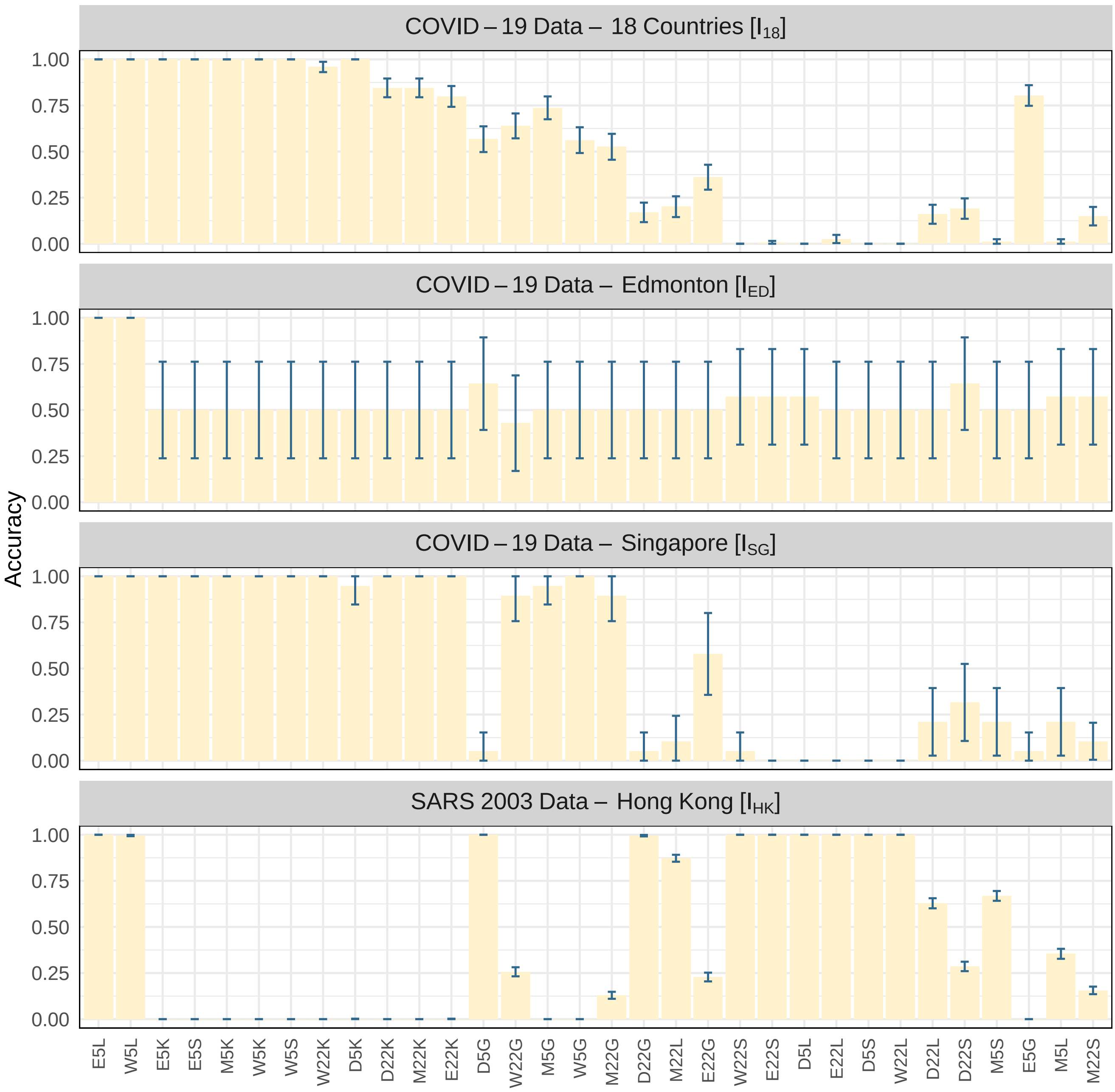

Figure 1: Classification accuracy of 32 synthetic-data-trained classifiers (horizontal axis, see Figure 2) to correctly classify subsequences of four empirical datasets. Error bars correspond to the 95% confidence intervals.

We also added the detailed results of COVID-19 data from Edmonton in the **Supplementary file** Table S22.

Comment 1.3

On that note, why not do the Mann-Whitney tests on the features obtained from the real data? Do you observe significant differences between outbreak vs. non-outbreak? Are they as one would expect based on dynamical systems theory? If not, maybe that is the issue here.

Response:

Thank you for the insightful suggestion. The Mann-Whitney U test can help to determine whether two sets of data are significantly different. However, in the case of the real-world datasets we used—namely, the COVID-19 data from Singapore (I_{SG}), the COVID-19 data from Edmonton, Canada (I_{ED}), and the SARS 2003 data from Hong Kong (I_{HK})—each dataset consists exclusively of either outbreak samples (labeled as "T") or non-outbreak samples (labeled as "N"), respectively. As a result, the Mann-Whitney U test cannot be applied to these datasets, as it requires data from two distinct groups to compare their distributions.

However, one of the additional datasets, the COVID-19 daily infection data from Edmonton, Canada (I_{ED}, #replicates = 14), includes both samples. A Mann-Whitney U test was conducted on I_{ED}, and we added the box plots and the following content to the **Supplementary file**:

9. Mann-Whitney U Test results over COVID-19 data from Edmonton, Canada

We conducted the Mann-Whitney U Test on the empirical data I_{ED} (COVID-19 data from Edmonton, Canada), which is the only empirical dataset that contains both labels. The results are presented in Figure S13 and Figure S14. Some features, such as features (13) and (14) from the 22 statistical features and the kurtosis from the 5 early warning signal indicators, exhibit statistically significant differences between the two labels. For some features with large p-values, we can still see differences with the box plots, likely due to the small sample size. Notably, the $p = 1$ and $p = 0.26$ for variance and AR1, respectively, suggest no significant difference between these two statistics across the two classes. This may explain the poor performance (AUC<0.5) on classification when using these two classic early warning signal indicators, as highlighted in Figure 2 of Chakraborty et al. [2].

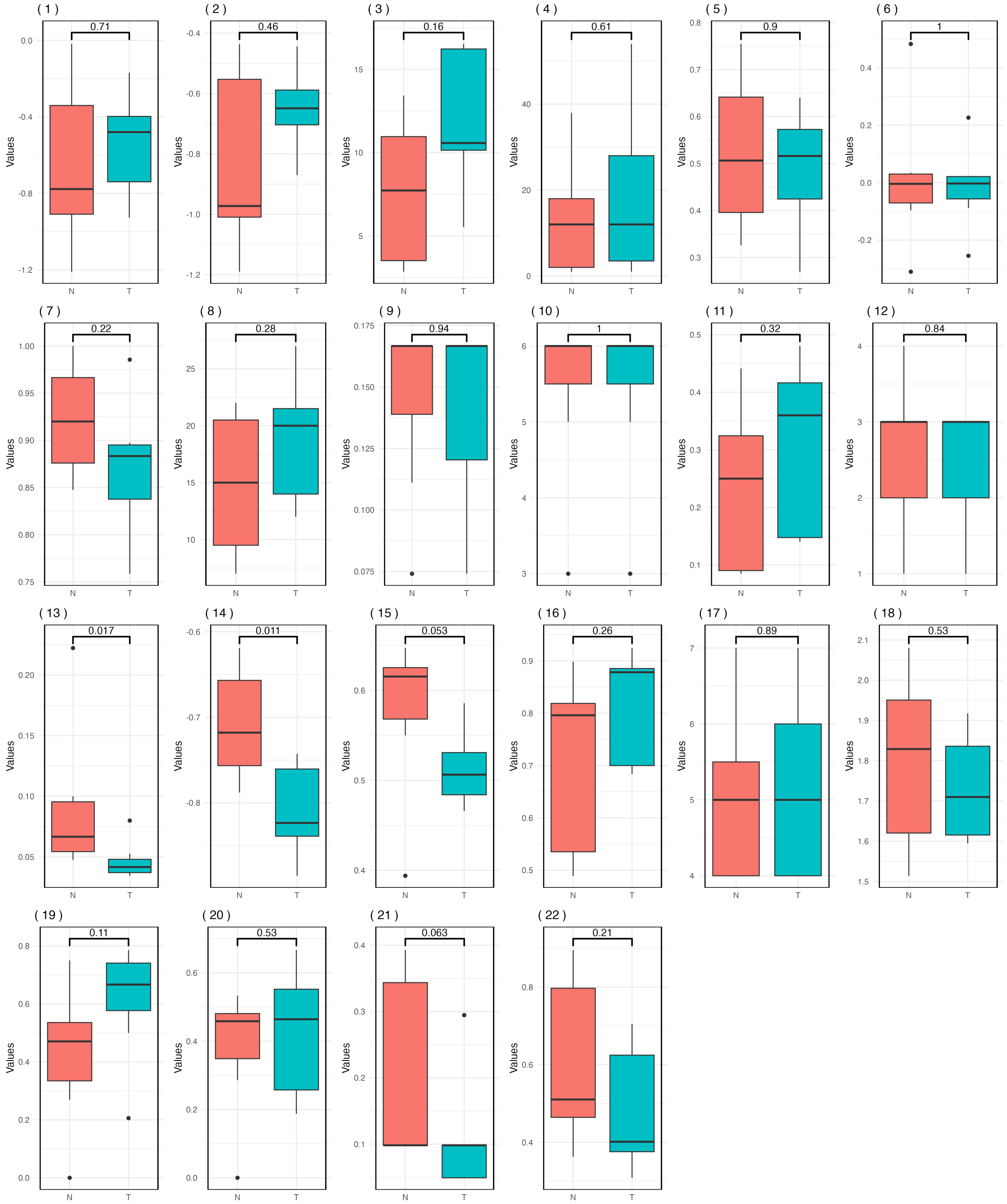

Figure 2: Features computed for I_{ED}. N indicates null bifurcation data and T indicates transcritical bifurcation data. P-Values for 22 Features using Mann-Whitney U Test.

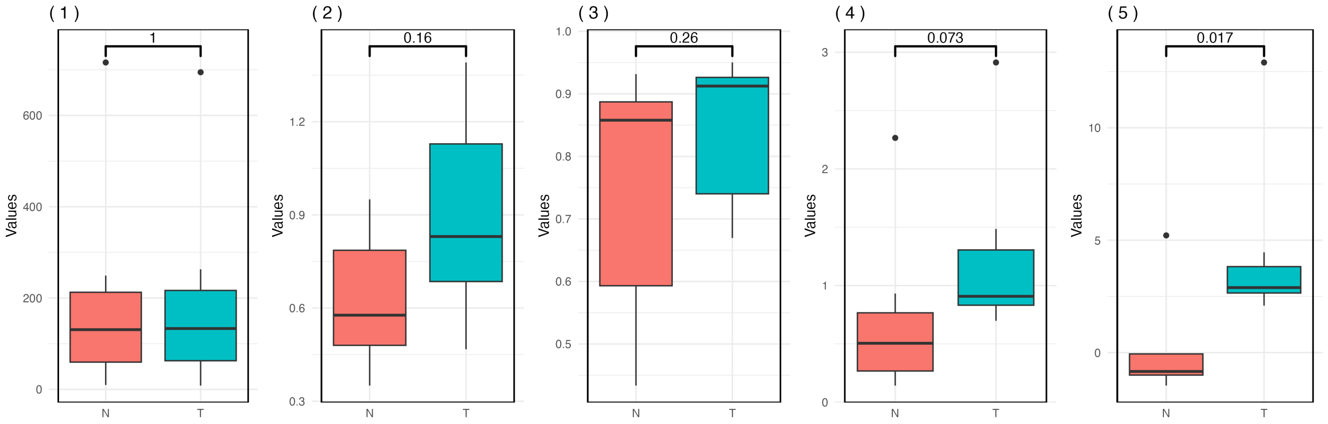

Figure 3: EWS indicators computed for I_{ED}. N indicates null bifurcation data and T indicates transcritical bifurcation data. P-Values for 5EWSI using Mann-Whitney U Test.

Comment 1.4

Personally, I find the analysis on the synthetic data interesting in itself, and a step forward in developing generalized machine learning approaches for predicting bifurcations. This alone could be an interesting publication in a more specialised journal. If the authors believe that this approach is effective on real data as it stands, then I will need more convincing.

Response:

We would like to again thank the reviewer for the encouraging feedback on the analysis of synthetic data. Rooted in the early warning signal theory, we applied machine learning to train the model to better predict outbreaks and non-outbreaks. Based on several choices of models for simulation, statistical feature extraction, and machine learning algorithms, we tested 32 models and found the same two best models to be used based on the consistently superior performance on 4 different empirical datasets. We believe that our work can contribute to the disease preparation, and is well-suited for publication in PLoS Computational Biology. Early warning signal theory, along with its applications in epidemiological studies, has been recognized as a powerful tool for integrating dynamic systems into machine learning predictions. Many related studies have been successfully published in PLoS Computational Biology [5, 12, 13].

We extended the empirical dataset to enhance the performance of certain models trained following the framework. We further use the COVID-19 daily data from Edmonton, Canada (I_{ED}), and a country-level dataset comprising daily COVID-19 data from 18 countries across five continents (I_{18}). The same two models perform well among all the empirical datasets. Specifically, these two models outperformed models trained by classic early warning signals (AUC<0.5), the deep learning model proposed by Bury. et al [1] (AUC<0.5), and Chakraborty. et al [2] model (AUC=0.71). Please refer to the revised manuscript, as elaborated in response to Comment 1.2, for further details regarding the effectiveness of this approach.

Since the same two models exhibit the best performance on two additional datasets, and these two models outperformed the previous models on the same dataset I_{ED}, we have more confidence to claim the effectiveness of these models in practice.

2 Response to reviewer 2

Comment 2.1

The work presented in this manuscript tests the hypothesis that a machine learning model based on features extracted from time series data can be used to forecast whether a transition point in an epidemic will occur. In general, this is an interesting approach which uses machine learning models trained on synthetic data and applies them to real world data.

Response:

We thank the reviewer for thoroughly reviewing the manuscript and appreciate the reviewer's feedback. Our responses to your comments are provided below. While this response letter includes the major revisions in blue with orange corresponding line numbers, minor changes making the revised manuscript self-contained are not included here to maintain the readability of the response letter. However, all changes are heightened in blue in the revised manuscript.

Comment 2.2

However, my main critique explained in several points below is that the authors train a wide array of models based on choices of features and the machine learning method, but it's not clear which model should be used in practice.

Response:

We thank the reviewer for pointing this out. In the following, we will explain why we have so many models and how we select the model.

Predicting novel infectious disease outbreaks has been a lingering challenge [14, 15], particularly in the early stages when data is limited, and phylogenetic analyses are unclear. In this work, we design a framework based on early warning signal (EWS) theory to predict infectious disease outbreaks and non-outbreaks. There are three configurations of the framework: (1) Model for simulation. To address the problem of insufficient data, we simulate the training data from the classic SIR model incorporating different types of noise to mimic the noise and fluctuation of the real world. (2) Feature extraction. According to the EWS theory, some statistics can serve as a precursor to indicate the upcoming transition such as disease outbreaks. With the simulation data, we extract two sets of statistical features, one comprising five basic early warning signal indicators (5EWSI) and the other one containing 22 complex features (22SF). (3) Machine learning algorithms. We apply four classic machine learning algorithms to train the models. As a result, we generate and evaluate 32 different models based on all the configuration combinations.

There are inherent discrepancies between the simulation and the real-world data. We have no prior knowledge of the novel disease and therefore we cannot determine the "best" choices for each configuration. Taking this into account, we do not expect all models to perform well on empirical datasets. Instead, performance on empirical datasets serves as the criterion for model selection and provides valuable insights into the optimal configurations for practical applications.

To make it more clear about the model selection, we have added the following to the ***1. Introduction***:

In the end, we test the classifiers on real-world COVID-19 incidence data and SARS data to validate their practical applicability. Performance on empirical datasets also serves as the criterion for model selection, as we tested multiple models derived from different framework configurations. (Line 128-130)

We further added the following to the ***2.4. Performance on empirical testing set*** of the manuscript:

Although our models perform well on withheld test sets, we need to further evaluate their practical utility using real-world datasets. Performance on empirical datasets is also the criterion for model selection as it provides insight into the optimal choices for the model of simulation, feature extraction, and the machine learning algorithm within the framework. (Line 216-219)

Comment 2.3

Uncertainty quantification would provide statistical confidence in the predictions, but this is missing from the study.

Response:

We would like to thank the reviewer for the suggestion, as the uncertainty quantification can enhance algorithmic transparency and trustworthiness [16]. Our work has accounted for uncertainties in different ways: (1) We included three types of noise in the SIR model for simulation to mimic the variability and unpredictable factors in real-world data. (2) We assumed the transmission rate parameter β to increase linearly with positive intercepts and slopes following triangular distribution, making the outbreak emergence time random. (3) We evaluated model performance by varying the sequence length and gap using rolling and expanding windows. (4) We tested the scenarios when data did not follow the homogeneity population assumption of the SIR model, and when data was highly underestimated. Results suggest that our models can handle these two scenarios. (5) Additionally, we also provided the 95 confidence interval for all the classification accuracies.

We revised the ***1. Introduction*** as follows:

Additionally, the dynamics in the real world are complex and coupled with exogenous and endogenous noise and stochasticity, unlike the deterministic model. To account for these uncertainties, we introduce three types of noise—white noise, multiplicative environmental noise, and demographic noise—to create three noise-induced SIR models for data simulation. (Line 102-105)

The time that the outbreak is triggered is random. Such randomness is also one of the uncertainties. We revised the ***4.2.1. Synthetic time series generation*** as follows:

Starting with a random initial value of $\beta(t = 0)$ such that $R_0(t = 0) < 1$, we gradually increase β over time to make R_0 exceed 1 to obtain the outbreak simulation. During this process, the presence of noise ensures that the infected individual I increases after the critical point. Without noise, I would become and remain zero when R_0 is initially less than 1. (Line 391-395)

Finally, we explored how uncertainties can affect the results by testing (1) how the model will perform when the real data is not consistent with the assumption of the SIR model; (2) how the model will perform when the real data is underestimated; and (3) how the model will perform when we have a shorter time series being classified.

We added the following to the ***2.4. Performance on empirical testing set*** to see the performance of data I_{18} that violates the model assumption:

Although violating the assumption of homogeneous population distribution in the SIR model, we utilize I_{18} to evaluate

the performance of classifiers on data that deviates from the model assumption. (Line 222-224)

For dataset I_{SG}, 17 out of 32 models performed better than random with 16 achieving an accuracy of 0.9 or higher. For dataset I_{18}, 18 models achieved an accuracy above 0.5, but only 9 models exceeded 0.9. Although I_{SG} and I_{18} are both national-level datasets, we observed generally lower accuracy on I_{18} compared to I_{SG}, which is consistent with our rationale that Singapore data is more suitable for SIR model assumptions. (Line 235-238)

The following is what we originally wrote in the ***2.4. Performance on empirical testing set*** to test how the performance will be influenced if we use shorter empirical data:

Although the minimum data length of I_{SG} and I_{18} is 14, we further access the performance of the classifiers on shorter replicates by removing 7 data points (i.e., ending the data one week before the outbreak timing) from these two COVID-19 datasets. Results presented in Supplementary Tables S25-S26 show that if the disease outbreak occurs seven days later, there is no significant difference between using the current data for prediction and additionally incorporating data from the next seven days. Thus, we can effectively predict the outbreak with a lead time of one week. It is worth noting that the "N" replicates are selected from the subset of SARS data with $R_e < 1$, with random lengths, hence we do not conduct the same test on shorter replicates for this dataset. (Line 247-254)

We should also acknowledge that asymptomatic or presymptomatic individuals exhibit scant influence on flu transmission [17], in contrast, COVID-19 incidence data are significantly underestimated due to these "unnoticeable" infections. We expand the empirical data on individuals infected with COVID-19 five-fold and repeat the experiment. **The results in Supplementary Tables S27 and S28 show that underestimation of incidence data does not have a significant impact on classification performance, no matter whether the original performance is excellent or undesirable.** (Line 254-259)

Here is what we added to the ***4.3. Empirical data used for testing*** to explain the empirical data I_{18} is underestimated:

I_{18} **(# replicates = 194; COVID-19 incidence data from 18 countries)** While the national data are often underestimated (due to presymptomatic and asymptomatic case) and against the homogeneity of the population, we still use the national-level data to test the robustness of the model. (Line 445-447)

We have addressed uncertainties to the best of our ability, and the classification results are the outcomes after considering these uncertainties. While theoretical analysis of uncertainties is not the primary focus of this work, we acknowledge the importance of uncertainty quantification (UQ) and have included the following in the ***3. Discussion***:

Finally, while simulations are used to enrich the training data for the models, inherent discrepancies exist between simulated and real-world data, potentially impacting the model's performance in practical scenarios. We therefore incorporate uncertainties into the simulation process by introducing various types of noise into the SIR model and randomizing the outbreak emergence times. Future research could further conduct theoretical analysis to examine how the noise and parameters can affect the classification outcomes using uncertainty quantification (UQ) [3]. (Line 335-340)

Comment 2.4

Given that the AUC scores are close to 1, this seems to strongly imply separability of the different classes based on the input features to the machine learning models. If this is the case, it would be useful to visualize this using dimensionality reductions techniques down to 2 or 3 dimensions, e.g., using PCA or t-SNE.

Response:

Thank you for the suggestion. Visualizing the data using t-SNE is indeed a valuable approach. We added the following to ***2.2 Performance on withheld testing set***:

Given the high AUC scores, we generated t-distributed stochastic neighbour embedding (t-SNE) plots (Supplementary Figure S10), which visually confirm the clear distinction between the two classes, supporting the near-perfect classification performance. (Line 158-161)

We added the following to **Supplementary file**:

4. t-SNE visualization of statistical features

To further assess the separability of the two classes based on input features, we generated t-distributed stochastic neighbour embedding (t-SNE) plots, shown in Supplementary Figure 4. These plots visually confirm the distinct separability of the two classes, supporting the near-perfect classification results on the withheld testing set.

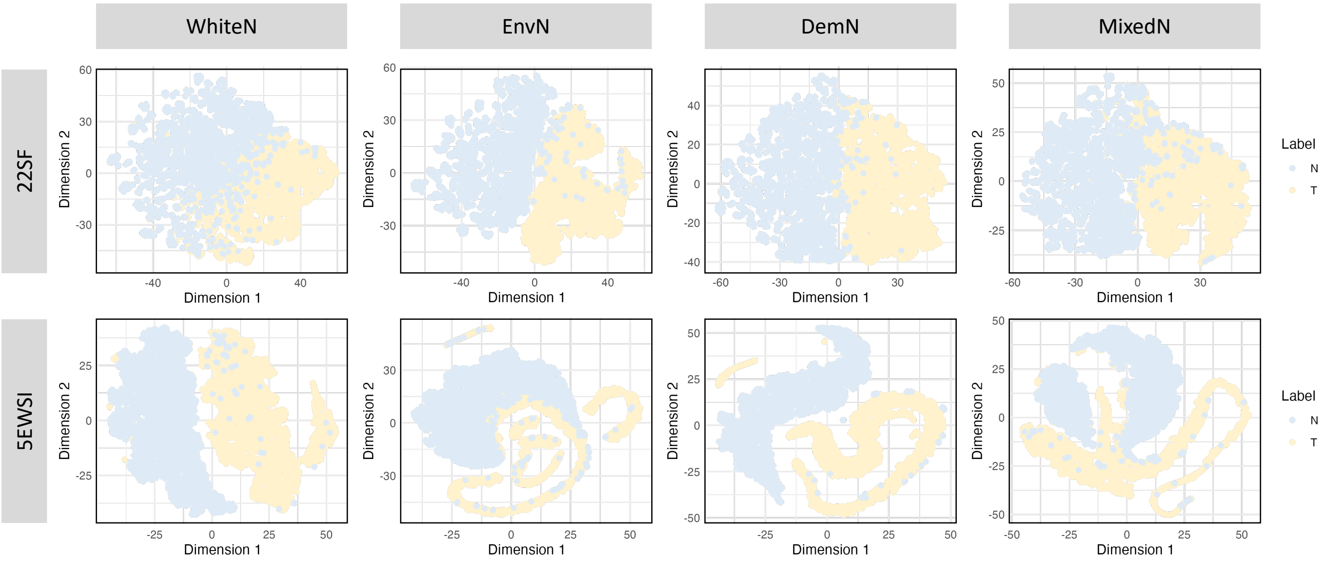

Figure 4: t-SNE visualization of statistical features derived from four datasets. Each panel contains a total of $12,000$ samples, with $6,000$ labeled as "T" and $6,000$ labeled as "N".

Comment 2.5

I think it would be helpful to plot the noiseless data with the noisy data, e.g., in Figure 1, to visualize how much noise is added to the data.

Response:

Thank you for the suggestion. In our simulation of the SIR model, the basic reproduction number (R_0) is initially set to $R_0 < 1$. Under these conditions, the spread of the infection slows down, leading to the eventual absence of infected individuals, i.e., $I = 0$. Once all infected individuals recover, no new infections will occur. Therefore, even if we linearly increase the transmission rate β such that $R_0 > 1$ at a random time point, the number of infected individuals I will remain at 0. However, by introducing noise into the system, I does not always remain 0, making it possible for the system to shift from a non-outbreak equilibrium to an outbreak equilibrium when β increases and R_0 exceeds 1.

We added the following text to clarify the reason we added the noise to the SIR model to ***4.2.1. Synthetic time series generation***:

Starting with a random initial value of $\beta(t = 0)$ such that $R_0(t = 0) < 1$, we gradually increase β over time to make R_0 exceed 1 to obtain the outbreak simulation. During this process, the presence of noise ensures that the infected individual I increases after the critical point. Without noise, I would become and remain zero when R_0 is initially less than 1. (Line 392-396)

We added the following to the ***4.2.2 Synthetic data input*** of the manuscript:

Simulations for both scenarios from each SDE model are visualized in Supplementary Figure S1. (Line 406-407)

We also added Figure 5 (Figure S1) to the **Supplementary file**.

1.2 Simulations with different amplitudes of noise

We plotted 20 randomly selected replicates of the simulation data for each scenario, with each replicate shown in a different shade in the corresponding panel. One can see that the simulation exhibits different shapes by adding different types of noise to the SIR model.

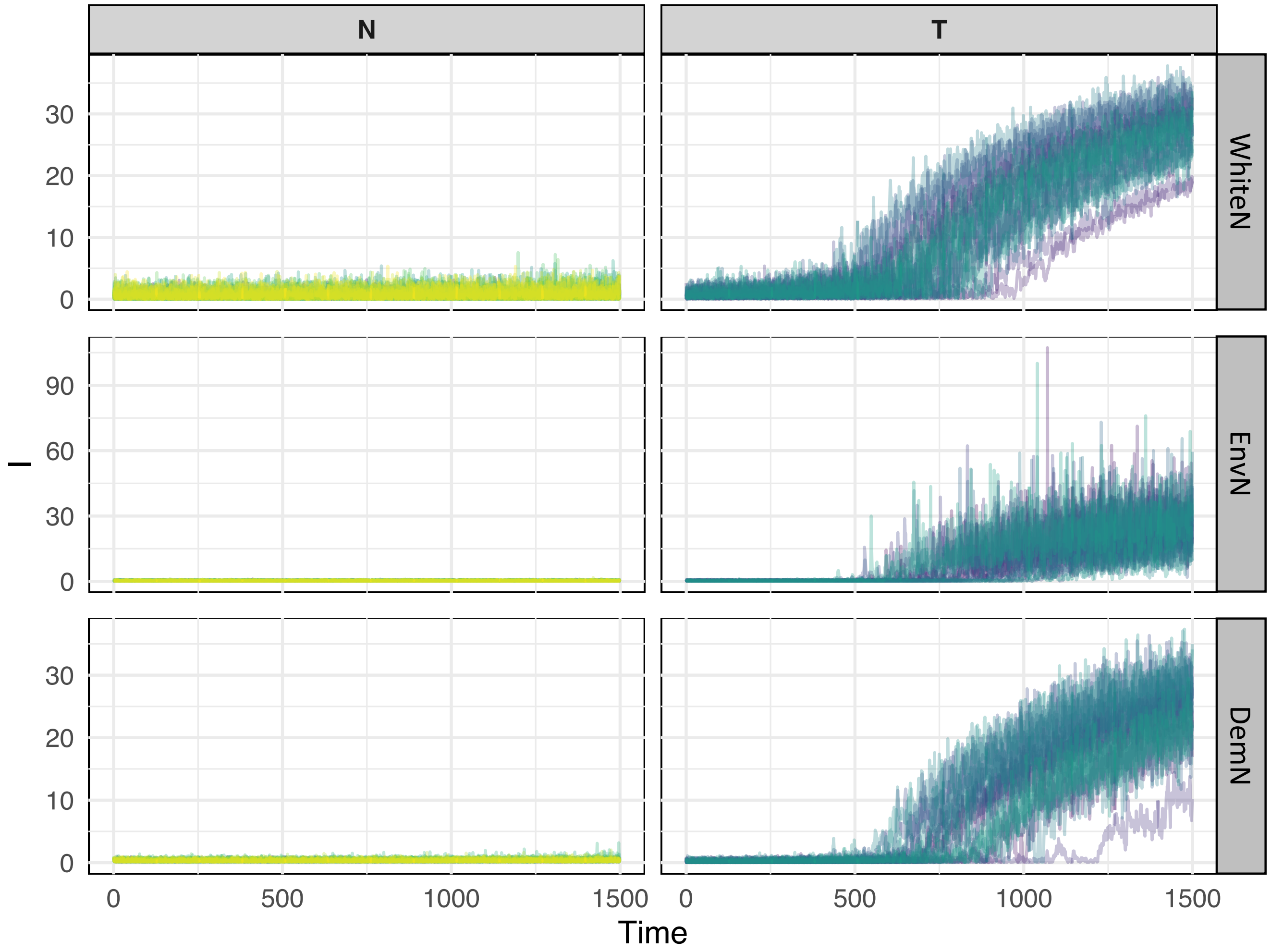

Figure 5: Simulation of infected individuals I of transcritical ("T") and null ("N") of the SIR model with white noise (WhiteN), multiplicative environmental noise (EnvN), and demographic noise (DemN).

Comment 2.6

A comparison to noiseless data for the results in Figure 3 would show whether adding noise affects the classification accuracy. I think Figure 3 shows that in some cases, because the AUC is exactly equal to 1, Demographic or White noise doesn't reduce classification accuracy.

Response:

We would like to clarify the rationale behind incorporating three distinct types of noise into the classic SIR model. As explained in Comment 2.5 above, without noise, it is impossible to simulate two distinct data scenarios for the training process.

Another reason for incorporating noise is to address the natural discrepancies between the SIR model and real-world conditions. While deterministic models, such as the SIR model used in this study, provide valuable insights into the transmission and recovery mechanisms of infectious diseases, these models are based on idealized conditions. In contrast, real-world infectious disease dynamics are inherently complex and influenced by random fluctuations [18]. Furthermore, real-world datasets often face challenges like variability, low quality, and reporting delays [19]. Adding noise to the SIR model allows it to better capture the inherent randomness of real-world scenarios, enhancing its applicability.

Given the limited prior knowledge available about new infectious diseases during their early stages, it is difficult to determine which type of noise would be more appropriate. Therefore, we incorporated additive white noise, multiplicative environmental noise, and demographic noise into the SIR model to generate the training data. While the model's performance on withheld data was satisfactory, with AUC scores close to or equal to 1, its performance on empirical data was notably degraded. This result is unsurprising, as uncertainties in real-world data can negatively impact model performance. We aim to identify a more suitable stochastic model, i.e. the SIR model with a specific type of noise, to improve training data generation and better apply it to empirical data. Based on the results presented in Figure 6 (Figure 6 in the revised manuscript), multiplicative environmental noise and additive white noise appear to be the most promising options.

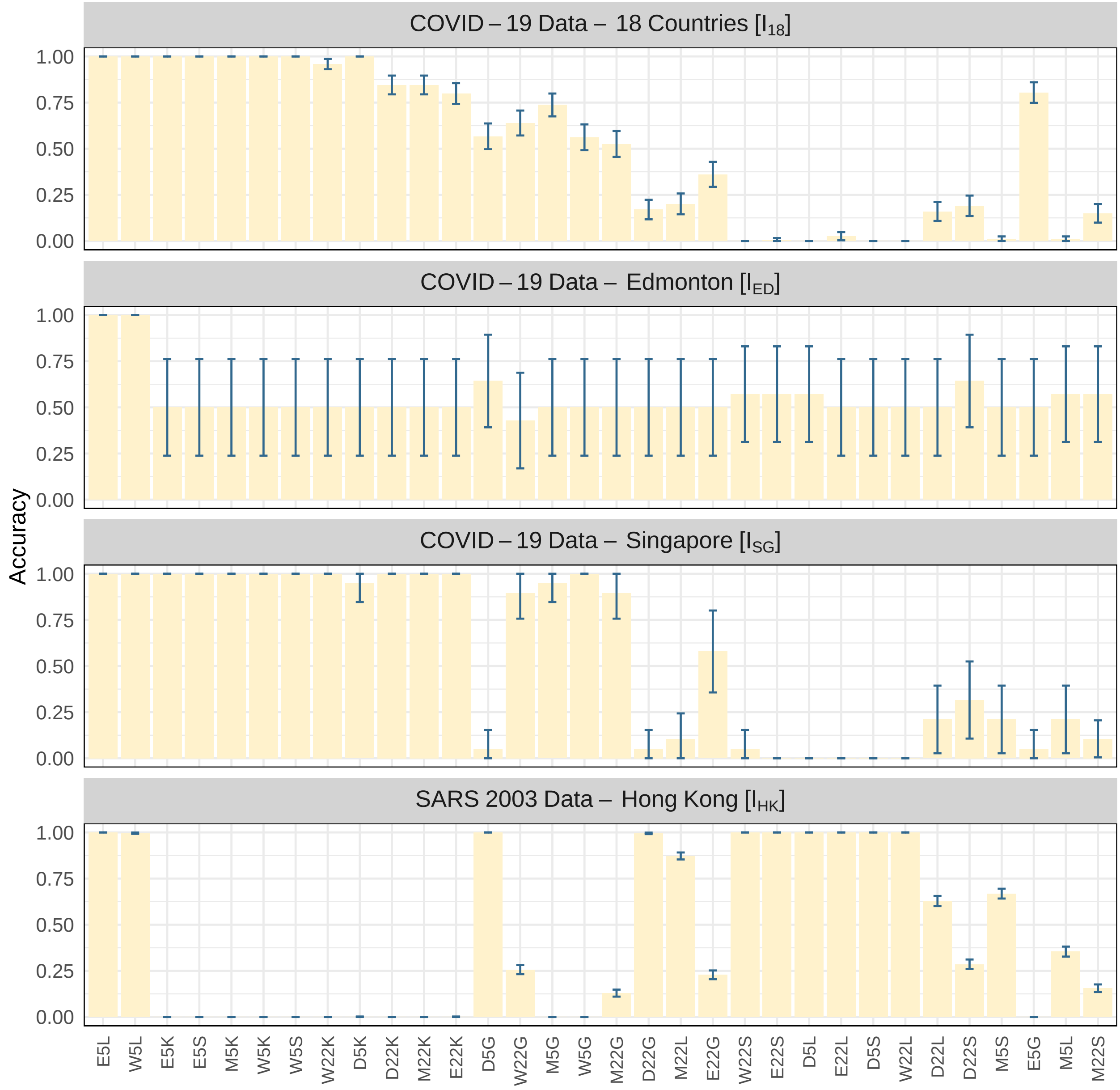

Figure 6: Classification accuracy of 32 synthetic-data-trained classifiers (horizontal axis, see Figure 2) to correctly classify subsequences of four empirical datasets. Error bars correspond to the 95% confidence intervals.

Comment 2.7

Figure 6 shows that the results between classifiers on real world data are heterogeneous. In practice, I don't think it would be possible to know a priori which classifier would be better to use. Can the authors discuss how can one choose the best classifier?

Response:

We agree with the reviewer that the results between classifiers on real-world data are indeed heterogeneous. This variation arises because the complex dynamics of real-world diseases are influenced by diverse environmental, social, and biological factors, which simulation data alone cannot fully capture. It is expected that the models would perform differently across these real-world data.

While it is easy to evaluate model performance in hindsight, predicting outcomes before a potential disease outbreak is far more challenging. In our study, the performance on empirical datasets serves as the criterion for model selection, offering valuable insights into the optimal configuration of our framework. In the original manuscript, we tested the models on two empirical datasets, identifying $E5L$ and $W5L$ as the best-performing models. In the revised version, we extended the evaluation to two additional empirical datasets. Notably, $E5L$ and $W5L$ consistently achieved superior accuracy across all four datasets, reinforcing our confidence in these models as the most effective options.

We've added the following to the ***1. Introduction*** to clarify how to choose the model:

In the end, we test the classifiers on real-world COVID-19 incidence data and SARS data to validate their practical applicability. Performance on empirical datasets also serves as the criterion for model selection, as we tested multiple models derived from different framework configurations. (Line 128-130)

We also added the following to the ***2.4. Performance on empirical testing set***

Although our models perform well on withheld test sets, we need to further evaluate their practical utility using real-world datasets. Performance on empirical datasets is also the criterion for model selection as it provides insight into the optimal choices for the model of simulation, feature extraction, and the machine learning algorithm within the framework. (Line 216-219)

Comment 2.8

Given that some classifiers achieve 1.0 accuracy, it would be informative if the authors showed the results of training a linear classifier model. In this case, the coefficients of the linear model could be used to interpret which features are driving the high accuracy.

Response:

Thank you for the suggestion on clarification and interpretability. We choose four classifier modes, namely (1) gradient boosting machine (GBM), (2) logistic regression model (LRM), (3) k-nearest neighbor (KNN), and (4) support vector machines (SVM). We have added the following to ***4.1. Time series classification and predictive models*** of the manuscript:

Among these, GBM and KNN are non-linear classifier models, LRM is a linear model, and a non-linear kernel was chosen for SVM. (Line 354-355)

In the **Supplementary file**, we have added Tables 1-2 (Table S6 and Table S7 in the supplementary file) to present the coefficients of logistic regression models using 22SF and 5EWSI, respectively.

Coefficients of logistic regression models (LRM) using 22SF and 5EWSI are presented in Table 1 and Table 2, respectively. Coefficients of variables vary significantly, meaning that these statistical features are different in simulations from different stochastic models.

No.	Feature	WhiteN	EnvN	DemN	MixedN
0	Intercept	23.972 22	−54.993 25	121.018 738	−5.577 65
1	DN_HistogramMode_5	−10.040 19	11.787 82	8.216 708	−0.443 00
2	DN_HistogramMode_10	7.037 75	−23.132 03	−2.405 667	1.391 35
3	CO_f1ecac	−0.333 88	−0.250 40	1.166 233	−0.872 48
4	CO_FirstMin_ac	−0.242 83	−0.077 06	−0.194 280	−0.183 38
5	CO_HistogramAMI_even_2_5	−59.315 63	−12.416 52	−89.935 070	−3.237 58
6	CO_trev_1_num	0.663 80	2.173 91	2.964 843	3.322 43
7	MD_hrv_classic_pnn40	19.142 38	−20.166 79	−21.127 921	8.082 51
8	SB_BinaryStats_mean_longstretch1	−0.118 36	0.413 44	−0.045 398	0.238 41
9	SB_TransitionMatrix_3ac_sumdiagcov	−19.402 97	−4.039 37	−51.055 396	−3.315 77
10	PD_PeriodicityWang_th0_01	−0.022 97	−0.020 08	0.005 524	−0.071 71
11	CO_Embed2_Dist_tau_d_expfit_meandiff	12.566 13	1.391 05	54.186 106	32.725 35
12	IN_AutoMutualInfoStats_40_gaussian_fmmi	−0.154 26	0.083 53	−0.142 763	0.089 74
13	FC_LocalSimple_mean1_tauresrat	−5.735 95	−0.864 14	−2.024 132	−1.023 23
14	DN_OutlierInclude_p_001_mdrmd	14.367 78	0.419 00	5.960 529	2.482 18
15	DN_OutlierInclude_n_001_mdrmd	−24.326 48	−14.596 58	−32.769 875	−17.037 70
16	SP_Summaries_welch_rect_area_5_1	21.715 80	−4.227 44	−19.625 003	8.712 33
17	SB_BinaryStats_diff_longstretch0	−0.064 63	1.354 61	0.426 635	1.159 49
18	SB_MotifThree_quantile_hh	−22.090 94	35.191 73	−30.977 310	−2.907 80
19	SC_FluctAnal_2_rsrangefit_50_1_logi_prop_r1	0.383 97	−0.769 98	0.662 704	−0.931 36
20	SC_FluctAnal_2_dfa_50_1_2_logi_prop_r1	−0.577 93	0.349 80	0.117 978	0.614 71
21	SP_Summaries_welch_rect_centroid	−14.365 07	−6.480 14	−12.646 018	−5.771 43
22	FC_LocalSimple_mean3_stderr	10.303 36	−14.184 61	−20.597 793	−3.435 73

Table 1: The coefficients of catch22 features of logistic regression models.

No.	Feature	WhiteN	EnvN	DemN	MixedN
0	Intercept	39.3243	6.605 14	−134.857	−4.974 55
1	Standard Deviation (SD)	4.8576	16.652 48	−7.332	−0.607 87
2	Coefficient of Variation (CV)	−116.8881	−13.946 75	266.171	−4.787 62
3	Autocorrelation at Lag1 (AR1)	30.2475	27.074 90	−8.878	25.923 54
4	Skewness	42.5173	−2.865 09	−51.229	3.622 73
5	Kurtosis	−4.4873	0.160 13	5.099	−0.177 10

Table 2: The coefficients of 5 early warning indicators of logistic regression models.

Comment 2.9

Similarly, the authors can report the results of a simple decision tree instead of a gradient boosting model, since the decision tree model will be more interpretable.

Response:

Thank you for the suggestion. We added the following to ***4.1. Time series classification and predictive models***

We additionally considered the simple decision tree (SDT) as it is more interpretable than GBM. However, SDT-trained models perform less effectively on withheld data than GBM-trained models, we therefore focus on the GBM. Detailed results of SDT, as well as the importance of features, are provided in Supplementary Table S5 and Figure S11. (Line 354-358)

We added the following in the **Supplementary file**:

5.1 Simple Decision Tree (SDT) and Gradient Boosting Machine (GBM)

While the simple decision tree (SDT) can provide more interpretable classification results, a more complex algorithm, the gradient boosting machine (GBM), can provide a higher classification accuracy as shown in Table 3.

**Evaluation**	**Model**	**22SF**			
		WhiteN	EnvN	DemN	MixedN
**Accuracy**	SDT	0.9867 (±0.0046)	0.9825 (±0.0052)	0.9958 (±0.0026)	0.9879 (±0.0044)
	GBM	0.9999 (±0.0004)	0.9991 (±0.0012)	1 (±0.0000)	0.9998 (±0.0006)
**Evaluation**	**Model**	**5EWSI**			
		WhiteN	EnvN	DemN	MixedN
**Accuracy**	SDT	0.9867 (±0.0046)	0.9646 (±0.0074)	0.9996 (±0.0008)	0.9738 (±0.0064)
	GBM	0.9988 (±0.0014)	0.9650 (±0.0074)	0.9996 (±0.0008)	0.9796 (±0.0057)

Table 3: Classification accuracy of Simple Decision Tree (SDT) and gradient boosting machine (GBM) on withheld testing sets.

5.2 Importance of statistical features

We recorded the importance of statistical features computed from the simple decision trees, as shown in Figure 7. For the 5EWSI features, AR1 and CV consistently exhibit high importance, aligning with expectations from EWS theory. Kurtosis, SD, and Skewness exhibit relatively similar values of importance across the four testing sets. There are only four features out of 22SF, namely CO_f1ecac (#3), FC_LocalSimple_mean3_stderr (#13), SP_Summaries_welch_rect_area_5_1 (#16), and SP_Summaries_welch_rect_centroid (#21), have uniformly high importance across four testing sets. The importance of other features varies depending on the type of testing set.

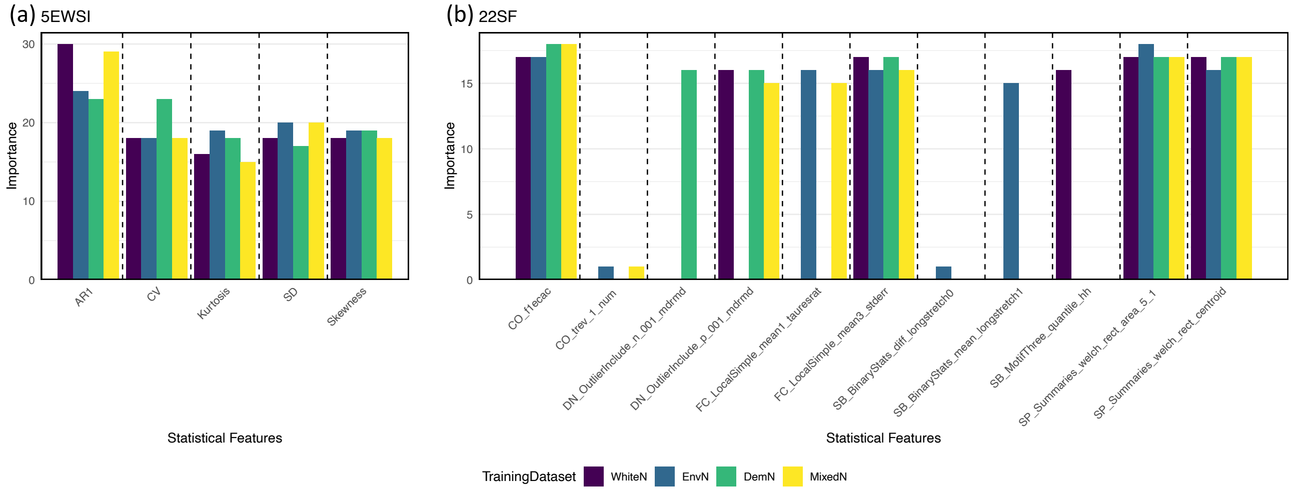

Figure 7: Importance of variables.

Comment 2.10

Can the authors provide uncertainty quantification for the machine learning model predictions? If not, how can one be confident in the prediction?

Response:

We thank the reviewer for pointing this out. We understand the importance of the reliability of models in practice. We have accounted for uncertainties as much as we could, as elaborated in response to Comment 2.3. The classification results are the outcomes after including these uncertainties, and theoretical analysis of uncertainties is not the primary focus of this work. Still, uncertainty quantification can be the future work to enhance the reliability of the prediction. We therefore added the following to the ***Discussion***:

Finally, while simulations are used to enrich the training data for the models, inherent discrepancies exist between simulated and real-world data, potentially impacting the model's performance in practical scenarios. We therefore incorporate uncertainties into the simulation process by introducing various types of noise into the SIR model and randomizing the outbreak emergence times. Future research could further conduct theoretical analysis to examine how the noise and parameters can affect the classification outcomes using uncertainty quantification (UQ) [3]. (Line 334-339)

In the original manuscript, we tested the models on two empirical datasets: COVID-19 daily data from Singapore (I_{SG}) and SARS data from Hong Kong (I_{HK}). There are two models, E5L and W5L, that exhibit best performance in both testing sets. To enhance the reliability of models for real-world data, we extended the empirical dataset to enhance the performance of certain models trained following the framework. We further use the COVID-19 daily data from Edmonton, Canada (I_{ED}), and a country-level dataset comprising daily COVID-19 data from 18 countries across five continents (I_{18}). As shown in Figure 6 of this response letter, the same two models perform well among all the empirical datasets. Specifically, these two models outperformed models trained by classic early warning signals (AUC<0.5), the deep learning model proposed by Bury. et al [1] (AUC<0.5), and Chakraborty. et al [2] model (AUC=0.71). Since the same two models exhibit the best performance on two additional datasets, and these two models outperformed the previous models on the same dataset I_{ED}, we have more confidence to claim the effectiveness of these models in practice.

We added the description of two more empirical datasets to ***4.3. Empirical data used for testing*** of the manuscript:

I_{ED} **(# replicates = 14; COVID-19 daily infection data from Edmonton, Canada)** The dataset I_{ED} was obtained from the City of Edmonton's Open Data Portal [6] and includes daily incidence data from March 2020 to July 2023. Similar to I_{SG}, the R_0 was estimated using `R` package `EpiEstim` [7], and subsequences with $R_0 < 0$ and length is larger than 56 (8 weeks) were selected. Note that this empirical dataset and the data processing are identical to the empirical datasets used in [2] for comparison purposes. (Line 440-444)

I_{18} **(# replicates = 194; COVID-19 incidence data from 18 countries)** While the national data are often underestimated (due to presymptomatic and asymptomatic case) and against the homogeneity of the population, we still use the national-level data to test the robustness of the model. To derive the replicates and the corresponding labels, we apply the R_e estimation from Huisman et al. [8] for COVID-19 incidence data, where the assumption is that the incidence data come from symptomatic individuals. Given the typical lag between disease occurrence and data collection, subsequences with lengths shorter than 14 days (i.e., two weeks), or replicates containing missing data are excluded. The final dataset comprises 194 replicates, labeled as "T", encompassing data from 18 countries. Detailed information on the sliced data is provided in Supplementary Table S20.

(Line 445-453)

We updated the ***Data availability*** of the manuscript:

Synthetic data are available at `https://doi.org/10.5281/zenodo.10967222` [9]. COVID-19 incidence data from Singapore and 18 countries is from Our World in Data [10]. The COVID-19 daily infection data comes from the City of Edmonton's Open Data Portal [6]. The SARS data for Hong Kong were obtained from `https://www.kaggle.com/datasets/imdevskp/sars-outbreak-2003-complete-dataset?resource=download` [11]. (Line 513-517)

Since we have two more empirical testing sets, we revised the ***2.4. Performance on empirical testing set***:

As shown in Figure 6, the performance varies across the four empirical datasets depending on the type of data used for model training, specifically the simulations generated from the SIR model with different types of noise. Notably, there are two models, $E5L$ and $W5L$, that consistently demonstrate high classification accuracy across all empirical datasets, making them the best-performing models among the 32 candidates. This result indicates that computing five classic early warning indicators from the data and using the logistic regression model would provide better classification performance.

For dataset I_{SG}, 17 out of 32 models performed better than random with 16 achieving an accuracy of 0.9 or higher. For dataset I_{18}, 18 models achieved an accuracy above 0.5, but only 9 models exceeded 0.9. Although I_{SG} and I_{18} are both national-level datasets, we observed generally lower accuracy on I_{18} compared to I_{SG}, which is consistent with our rationale that Singapore data is more suitable for SIR model assumptions. The performance of I_{18}, I_{SG} and I_{HK}, or in other words, between replicates solely labeled as "T" (outbreaks) or "N" (non-outbreaks) seem to be complementary. Most classifiers performing well in classifying "T" replicates fail to predict replicates labeled as "N", except for two classifiers, E5L and W5L, achieving an accuracy of 1 (± 0.0000) in out-of-sample empirical data classification tasks. For dataset I_{ED}, which is also used in Chakraborty et al. [2] as the empirical testing set, the best two models (AUC = 1) outperformed the models trained by classic early warning signals (AUC $<$ 0.5), the deep learning model proposed by Bury. et al [1] (AUC $<$ 0.5), and Chakraborty. et al [2] model (AUC = 0.71). Note that the error bar for I_{ED} is significantly larger compared to the other three datasets as its sample size is much smaller. (Line 228-245)

Comment 2.11

Can the authors provide an explanation/interpretation for why the different types of noise yield different results in figure 7?

Response:

Thank you for the opportunity to clarify. As there are six figures in the original manuscript, we assume the reviewer is referring to Figure 6. The models were trained exclusively on simulated data, as sufficient data on novel diseases is typically unavailable during the early stages for training purposes. The observed variation in performance on empirical data arises because the dynamics of infectious diseases in the real world are inherently complex and influenced by randomness from various sources. Given the nature of this randomness is unknown, we investigated three types of noise commonly used in previous studies to approximate such variability. Based on the performance of models on real-world data, we can identify the noise type(s) that most effectively capture this complexity, allowing us to select the optimal configuration for our framework.

We've revised the ***2.4. Performance on empirical testing set*** as follows:

As shown in Figure 1, the performance varies across the four empirical datasets depending on the type of data used for model training, specifically the simulations generated from the SIR model with different types of noise. Notably, there are two models, $E5L$ and $W5L$, that consistently demonstrate high classification accuracy across all empirical datasets, making them the best-performing models among the 32 candidates. These findings suggest that simulating the SIR model with either white noise or environmental noise is preferable. Additionally, computing five classic early warning indicators from the data and using the logistic regression model would provide better classification performance. (Line 228-234)

Comment 2.12

Model E5L has one of the lowest AUCs in figure 3, and hence one would assume it wouldn't perform well on real data. However, it has the highest accuracy in figure 6 when testing on real data. How can this be explained?

Response:

We thank the reviewer for pointing this out. While the model $E5L$ demonstrates the second-lowest AUC on the withheld testing set, as illustrated in Fig. 3 (Figure 3 in the manuscript), it still achieves an impressive AUC of 0.9938 ± 0.0032. It is worth noting that the y-axis in Fig. 3 spans from 0.9850 to 1, which underscores the near-perfect performance of all classifiers on the withheld testing sets. For this reason, we cannot determine which model should be used. We further conducted experiments on empirical datasets, aiming to assess their practical applicability and identify the most suitable model based on performance in real-world scenarios. As shown in Fig. 6 (Figure 6 in the manuscript), the performance of the models on four empirical datasets reveals that $E5L$ and W5L consistently outperform the other models across all datasets. These results suggest that these two models are strong candidates for model selection, as they perform well on both synthetic (withheld) and all empirical datasets.

References

[1] Thomas M Bury, RI Sujith, Induja Pavithran, Marten Scheffer, Timothy M Lenton, Madhur Anand, and Chris T Bauch. Deep learning for early warning signals of tipping points. *Proceedings of the National Academy of Sciences*, 118(39):e2106140118, 2021.

[2] Amit K Chakraborty, Shan Gao, Reza Miry, Pouria Ramazi, Russell Greiner, Mark A Lewis, and Hao Wang. An early warning indicator trained on stochastic disease-spreading models with different noises. *Journal of the Royal Society Interface*, 21(217):20240199, 2024.

[3] Moloud Abdar, Farhad Pourpanah, Sadiq Hussain, Dana Rezazadegan, Li Liu, Mohammad Ghavamzadeh, Paul Fieguth, Xiaochun Cao, Abbas Khosravi, U Rajendra Acharya, et al. A review of uncertainty quantification in deep learning: Techniques, applications and challenges. *Information fusion*, 76:243–297, 2021.

[4] Suzanne M O'Regan and John M Drake. Theory of early warning signals of disease emergenceand leading indicators of elimination. *Theoretical Ecology*, 6:333–357, 2013.

[5] Daniele Proverbio, Françoise Kemp, Stefano Magni, and Jorge Gonçalves. Performance of early warning signals for disease re-emergence: A case study on covid-19 data. *PLOS Computational Biology*, 18(3):e1009958, 2022.

[6] City of Edmonton's Open Data Portal. Covid-19 in edmonton: Daily active cases. `https://data.edmonton.ca/Community-Services/COVID-19-in-Edmonton-Daily-Active-Cases/qkyj-dqjp`, 2024.

[7] Anne Cori, Simon Cauchemez, Neil M Ferguson, Christophe Fraser, Elisabeth Dahlqwist, P Alex Demarsh, Thibaut Jombart, Zhian N Kamvar, Justin Lessler, Shikun Li, et al. Package 'epiestim'. *CRAN: Vienna Austria*, 2020.

[8] Jana S Huisman, Jérémie Scire, Daniel C Angst, Jinzhou Li, Richard A Neher, Marloes H Maathuis, Sebastian Bonhoeffer, and Tanja Stadler. Estimation and worldwide monitoring of the effective reproductive number of sars-cov-2. *Elife*, 11:e71345, 2022.

[9] Shan Gao. Code and Data for Early detection of disease outbreaks and non-outbreaks, April 2024. Zenodo, doi: 10.5281/zenodo.10967222, `https://zenodo.org/records/10967222`.

[10] Edouard Mathieu, Hannah Ritchie, Lucas Rodés-Guirao, Cameron Appel, Charlie Giattino, Joe Hasell, Bobbie Macdonald, Saloni Dattani, Diana Beltekian, Esteban Ortiz-Ospina, and Max Roser. Coronavirus pandemic (covid-19). *Our World in Data*, 2020. https://ourworldindata.org/coronavirus.

[11] Devakumar K. P. Sars 2003 outbreak dataset, 2020.

[12] Steven J Lade and Thilo Gross. Early warning signals for critical transitions: a generalized modeling approach. *PLoS computational biology*, 8(2):e1002360, 2012.

[13] Emma Southall, Michael J Tildesley, and Louise Dyson. Prospects for detecting early warning signals in discrete event sequence data: Application to epidemiological incidence data. *PLoS computational biology*, 16(9):e1007836, 2020.

[14] Edward C Holmes, Andrew Rambaut, and Kristian G Andersen. Pandemics: spend on surveillance, not prediction, 2018.

[15] Samuel V Scarpino and Giovanni Petri. On the predictability of infectious disease outbreaks. *Nature communications*, 10(1):898, 2019.

[16] Venkat Nemani, Luca Biggio, Xun Huan, Zhen Hu, Olga Fink, Anh Tran, Yan Wang, Xiaoge Zhang, and Chao Hu. Uncertainty quantification in machine learning for engineering design and health prognostics: A tutorial. *Mechanical Systems and Signal Processing*, 205:110796, 2023.

[17] Eleni Patrozou and Leonard A Mermel. Does influenza transmission occur from asymptomatic infection or prior to symptom onset? *Public health reports*, 124(2):193–196, 2009.

[18] Lars Hufnagel, Dirk Brockmann, and Theo Geisel. Forecast and control of epidemics in a globalized world. *Proceedings of the national academy of sciences*, 101(42):15124–15129, 2004.

[19] Caroline O Buckee, Maria IE Cardenas, June Corpuz, Arpita Ghosh, Farhana Haque, Jahirul Karim, Ayesha S Mahmud, Richard J Maude, Keitly Mensah, Nkengafac Villyen Motaze, et al. Productive disruption: opportunities and challenges for innovation in infectious disease surveillance. *BMJ global health*, 3(1):e000538, 2018.
